# Stabilizing the framework of SAPO-34 zeolite toward long-term methanol-to-olefins conversion

**DOI:** 10.1038/s41467-021-24403-2

**Published:** 2021-08-02

**Authors:** Liu Yang, Chang Wang, Lina Zhang, Weili Dai, Yueying Chu, Jun Xu, Guangjun Wu, Mingbin Gao, Wenjuan Liu, Zhaochao Xu, Pengfei Wang, Naijia Guan, Michael Dyballa, Mao Ye, Feng Deng, Weibin Fan, Landong Li

**Affiliations:** 1 School of Materials Science and Engineering, and National Institute for Advanced Materials, Nankai University, Tianjin, P.R. China; 2 Key Laboratory of Advanced Energy Materials Chemistry of the Ministry of Education, Collaborative Innovation Center of Chemical Science and Engineering, Nankai University, Tianjin, P.R. China; 3 National Center for Magnetic Resonance in Wuhan, State Key Laboratory of Magnetic Resonance and Atomic and Molecular Physics, Key Laboratory of Magnetic Resonance in Biological Systems, Wuhan Institute of Physics and Mathematics, Chinese Academy of Sciences, Wuhan, P. R. China; 4 Dalian Institute of Chemical Physics, Chinese Academy of Sciences, Dalian, P. R. China; 5 State Key Laboratory of Coal Conversion, Institute of Coal Chemistry, Chinese Academy of Sciences, Taiyuan, Shanxi P. R. China; 6 Institute of Chemical Technology, University of Stuttgart, Stuttgart, Germany

**Keywords:** Catalytic mechanisms, Heterogeneous catalysis

## Abstract

As a commercial MTO catalyst, SAPO-34 zeolite exhibits excellent recyclability probably due to its intrinsic good hydrothermal stability. However, the structural dynamic changes of SAPO-34 catalyst induced by hydrocarbon pool (HP) species and the water formed during the MTO conversion as well as its long-term stability after continuous regenerations are rarely investigated and poorly understood. Herein, the dynamic changes of SAPO-34 framework during the MTO conversion were identified by 1D ^27^Al, ^31^P MAS NMR, and 2D ^31^P-^27^Al HETCOR NMR spectroscopy. The breakage of T-O-T bonds in SAPO-34 catalyst during long-term continuous regenerations in the MTO conversion could be efficiently suppressed by pre-coking. The combination of catalyst pre-coking and water co-feeding is established to be an efficient strategy to promote the catalytic efficiency and long-term stability of SAPO-34 catalysts in the commercial MTO processes, also sheds light on the development of other high stable zeolite catalyst in the commercial catalysis.

## Abstract (web summary)

Stability of zeolite catalysts is a highly desirable property for commercial methanol to olefins conversion but extremely challenging to achieve. Here, the authors combine the catalyst pre-coking and water co-feeding to develop an efficient strategy to enhance the long-term stability of SAPO-34 catalyst.

## Introduction

As a sustainable route to obtain lower olefins, methanol-to-olefin (MTO) conversion has attracted broad attention during the past decades^1–19^. Up to now, more than 20 MTO units with an olefin capacity of >10 Mt per year have been operated in China^20,21^. SAPO-34 zeolite with suitable acidity and pore structure has been utilized as a commercial MTO catalyst, and a remarkable catalytic activity with a high yield of lower olefins (>85%) could be achieved^20^. However, a rapid deactivation owing to the bulk coke compound formation requires a continuous regeneration of the catalyst and a fluidized bed reactor combined with a regenerator is employed in the commercial operation^21^. After long-term running, a slight decrease of the catalytic efficiency will occur due to the catalyst loss and the partial damage of the catalyst; therefore, a regular supplement of the catalyst is required to maintain the stable yield of C_2_ and C_3_ olefins in the commercial process. Thus, an enhancement of the long-term stability of SAPO-34 catalyst is still a challenge for the development of an excellent commercial MTO catalyst.

As is known, SAPO-34 zeolite possesses good hydrothermal stability. However, with the presence of water, the structure of zeolites is labile and a reversible hydrolysis of T-O-T bonds can occur even at room temperature^22–24^. A recent report also indicated that the reversible hydrolysis of T-O-T bonds in SAPO-34 zeolite can occur at higher temperatures of 273–673 K^25^. In addition, after longtime exposure to moisture at room temperature, the irreversible hydrolysis of Si-O-Al bonds with partial framework degradation would occur eventually^22,26,27^. To enhance the stability of SAPO-34 zeolite for longtime preservation, several methods have been utilized, e.g., coverage of Brønsted acid sites (BASs) with ammonia^28,29^, reducing the surface defects with postsynthetic silylation treatment^30,31^ or self-defect healing^32^, and high-temperature steaming for decreasing the BAS density of zeolites^33^.

MTO conversion is a typical acid-catalyzed reaction. The active intermediates, e.g., polymethylbenzenes and the corresponding carbenium ions, can be formed at the BAS and induce the MTO conversion. On the other hand, coke compounds are rapidly formed at the BAS, causing the catalyst deactivation. Recent studies have demonstrated that the formation of hydrocarbon pool (HP) species during the MTO conversion could induce a unit cell expansion of the zeolite catalysts^34,35^ and the active HP species have a spatial interaction/proximity with the aluminum sites in the framework of the zeolite^36,37^. Detailed information on structure changes, e.g., of the T-O-T bonds in SAPO-34 zeolite, induced by HP species during the MTO conversion, however, is still missing. In addition, recent theoretical simulations indicated that water molecules formed during the MTO conversion can preferentially adsorb on the BAS compared with the formed alkenes and have an equal probability to compete at the BAS under reaction conditions^38,39^. Therefore, an enhanced catalyst lifetime with a lower coking rate during the MTO conversion could be obtained after water co-feeding, because the BAS are partially occupied by water molecules^38,40–42^. However, the structure changes of SAPO-34 catalysts induced by the in situ produced or co-fed water during the MTO conversion, especially for the catalysts after repeated regenerations, are not involved in current researches, which are extremely important for developing SAPO-34 materials with long-term stability in the commercial MTO process.

In the present work, we successfully identify the dynamic changes of the SAPO-34 framework induced by HP species and water formed during the MTO conversion based on one-dimensional (1D) ^27^Al, ^31^P magic-angle spinning nuclear magnetic resonance (MAS NMR), and two-dimensional (2D) ^31^P-^27^Al HETCOR (HETeronuclear CORrelation) NMR spectroscopy. For the first time, it was found that a reversible unit cell expansion with the increase of the mean P-O-Al bond angles in SAPO-34 zeolite catalysts occurred with the progress of MTO conversion. In addition, the breakage of T-O-T bonds in SAPO-34 catalysts after longtime exposure to moisture or continuously repeated regenerations in the MTO conversion could be efficiently suppressed by coke formation. The combination of catalyst pre-coking and water co-feeding is established to be the efficient strategy to enhance both the single-pass lifetime and long-term stability of SAPO-34 catalysts in the commercial MTO processes.

## Results

### MTO conversion over SAPO-34 catalysts

**Fig. 1:**
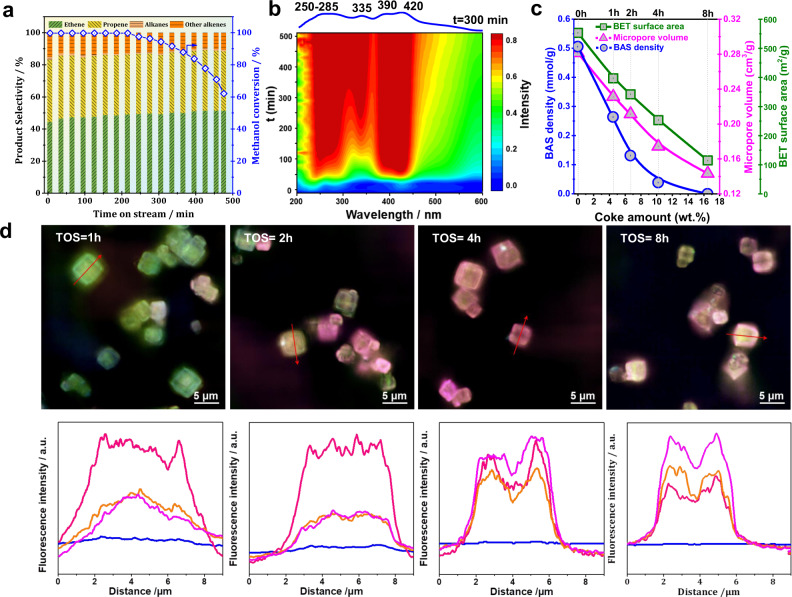
Catalytic performance, organic intermediates, and textural properties of SAPO-34 catalysts. Methanol conversion and product distribution during the MTO conversion over SAPO-34 catalyst at 673 K (**a**). In situ UV-vis spectra of MTO conversion over SAPO-34 recorded at 673 K (**b**). In **c**, the number of accessible Brønsted acid sites, the BET surface area, and micropore volume of the SAPO-34 catalyst during the MTO conversion at 673 K are plotted as a function of coke amounts. In **d**, the spatiotemporal distribution of carbonaceous species obtained from SIM in SAPO-34 samples during the MTO conversion at 673 K for different TOS of 1–8 h. The fluorescence intensities of the selected lines are also given. The SIM pictures are the fluorescence originating from the overlap of four curves with different laser excitations of 405 nm (detection at 435–485 nm, blue curve), 488 nm (detection at 500–545 nm, pink curve), 561 nm (detection at 570–640 nm, orange curve), and 640 nm (detection at 663–738 nm, magenta curve).

Silicoaluminophophate SAPO-34 zeolite with a *n*_Si_/(*n*_Al_ + *n*_Si_ + *n*_P_) ratio of 0.05 was utilized as a model MTO catalyst (Supplementary Table S1 and Fig. 1). In general, sub-complete methanol conversion would be more suitable for the kinetic experiments of the catalyst deactivation^43,44^. However, we aim to compare the long-term stability during the continuous regenerations under the similar conditions of commercial MTO processes and, therefore, full methanol conversion was utilized in the present study. The catalytic performance of SAPO-34 catalyst during the MTO conversion at 673 K with a time-on-stream (TOS) of 8 h was shown in Fig. 1a. A slight deactivation occurs when increasing the TOS of >210 min. Simultaneously, large amounts of neutral polyalkylaromatics and the corresponding carbo-cations giving rise to the ultraviolet-visible (UV-vis) bands at 260–300 and 370–390 nm, respectively, already start to occur in the initial period of MTO conversion (Fig. 1b). With the further progress of MTO conversion, dienylic carbenium ions (335–340 nm) and neutral polyaromatics species and/or methylated naphthalene carbo-cations (425–430 nm) were formed as the dominant organic species. These large organic species could block the micropore and hinder the accessibility of BASs to methanol molecules, causing catalyst deactivation. This can be well-supported by the results of Brunauer–Emmett–Teller (BET) surface area and the microporous volume plotted as a function of the coke amounts as shown in Fig. 1c. In addition, although no accessible BASs (*δ*_1H_ = 6.2 p.p.m.) were detected at 673 K with a TOS of 8 h (Fig. 1c and Supplementary Figs. 2 and 3), a few amounts of benzene-based carbenium ions (*δ*_1H_ = 5.1 p.p.m.) were still available to trigger the MTO conversion and^45^, therefore, obvious methanol conversion could be still achieved (Fig. 1a). However, with the complete absence of accessible BASs and benzene-based carbenium ions, the catalyst was totally deactivated^45,46^. For investigating the structure changes of this SAPO-34 catalyst during the MTO conversion, different reaction temperatures of 573 and 623 K were also employed for comparison, and similar information as aforementioned could be obtained (see Supplementary Figs. 4–8).

To further investigate the spatiotemporal evolution of organic compounds formed during the MTO conversion, the high-resolution structured illumination microscopy (SIM) was also employed. Following the previous research results^17,47–51^, the characteristic UV-vis signals of benzene- (B^+^) and naphthalene-based (N^+^) carbenium ions should appear at about 390 and 480 nm, respectively, and those of phenanthrene- (PH^+^) and pyrene-based (PYR^+^) carbenium ions appear at ~560 and ~640 nm, respectively. Thereupon, distinguishable emission wavelengths in the range of 480–490, 500–520, 620–630, and 670–700 nm are expected for the aforementioned carbenium ions, respectively. For deeper insight into the spatiotemporal distribution of the organic compounds within the zeolite crystals, the excitation and emission wavelengths of the aromatic-based carbenium ions are fully covered in the present SIM experiments. As shown in Fig. 1d, the carbonaceous species start to form in the crystal center and, thereafter, expand from the center to the rim. In addition, large amounts of N^+^, which act as the active HP species, have already formed within the first hour. With the progress of MTO conversion, the HP species gradually transfer to coke precursors in the crystal center. Accordingly, the fluorescence intensities of HP species at the crystal center are lower than that at the rim. When catalyst deactivation occurs, more coke precursors are observed at the crystal rim, which can block the micropore and hinder methanol to access the acid sites, eventually causing catalyst deactivation, in line with the aforementioned results in Fig. 1c.

Recent reports have demonstrated that the HP species formed during the MTO conversion have a spatial interaction/proximity with the BAS and could induce the unit cell expansion of the zeolite catalysts^34–37^. The missing information on the structure changes of SAPO-34 zeolites during the MTO conversion under various reaction conditions will be discussed hereinafter.

### Hydrocarbon- and water-induced dynamic structure changes of the SAPO-34 catalyst

It is well acknowledged that the active HP species, e.g., polymethylbenzenes and the corresponding carbenium ions, can be formed at the BAS of zeolite catalysts during the MTO conversion^52–54^. Therefore, the dynamic structure changes of SAPO-34 catalysts induced by HP species during the MTO conversion were first investigated by ^27^Al and ^31^P MAS NMR spectroscopy. For the used SAPO-34 catalyst after the MTO conversion at different temperatures for TOS of 1 h, similar ^27^Al MAS NMR spectra with a dominant signal at about 32.5 p.p.m. due to the tetrahedrally coordinated framework Al (Al^IV^) atoms occur (Supplementary Fig. 9a)^55^. In the ^31^P MAS NMR spectra, the chemical shifts of the tetrahedrally coordinated P (P^IV^) atoms at −30.1 p.p.m.^56^ slightly move to the high-field direction of −30.3 p.p.m. in comparison with the fresh sample (Supplementary Fig. 10a). According to previous studies^57–59^, the chemical shifts (*δ*) of P^IV^ atoms in AlPO_4_-zeolites correlate with the mean P-O-Al bond angle (*α*): *α*  = −0.802 * *δ* + 124. Therefore, slight changes of these ^31^P MAS NMR signals could be explained with the gradual formation of HP species inducing a unit cell expansion of the zeolite catalyst, thus causing a slight increase of the mean P-O-Al bond angle with the progress of MTO conversion.

**Fig. 2:**
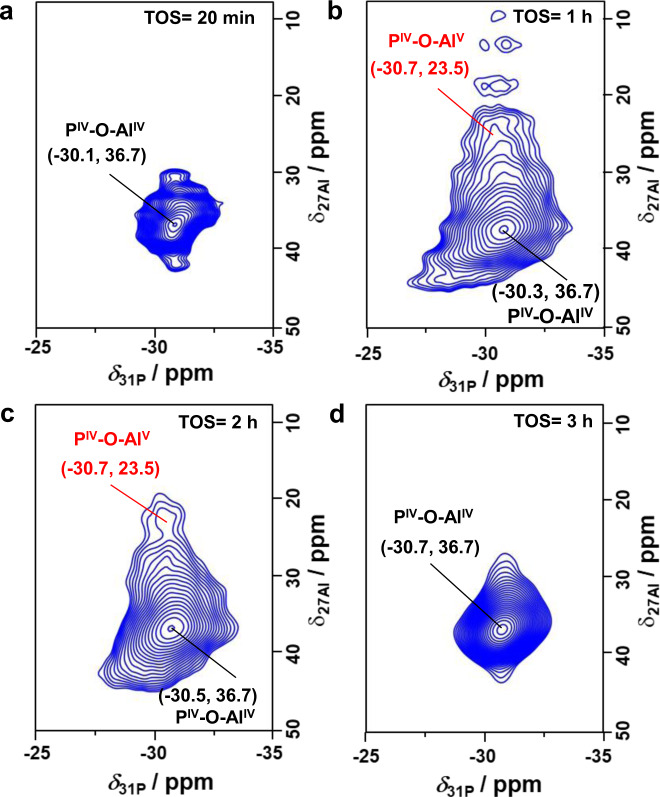
Discrimination of ^31^P-^27^Al correlations by 2D ^31^P-^27^Al HETCOR MAS NMR experiments. **a**–**d** 2D ^31^P-^27^Al HETCOR MAS NMR spectra of the spent SAPO-34 catalysts after MTO conversion at 623 K for different TOS of 20 min to 3 h.

**Fig. 3:**
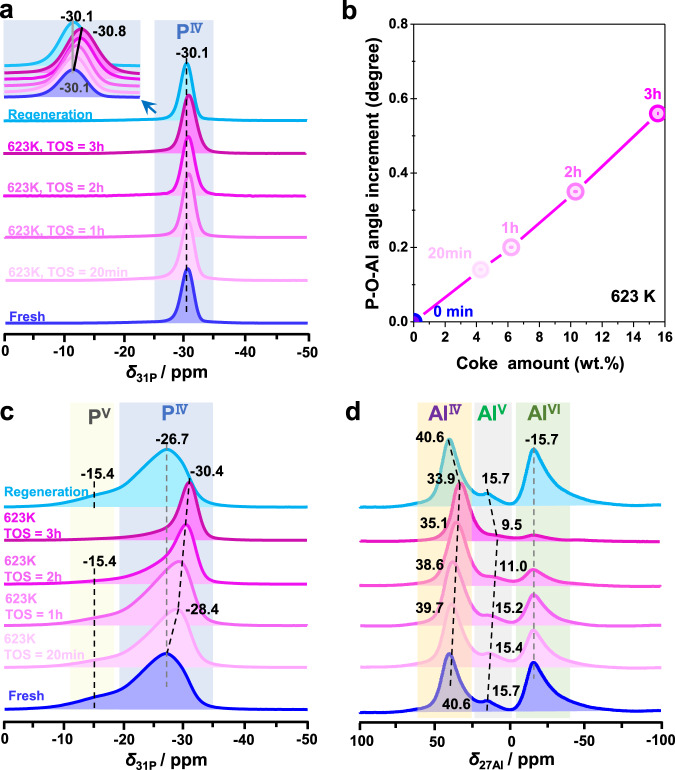
Probing structural dynamic changes of SAPO-34 catalysts by 1D ^27^Al and ^31^P MAS NMR spectroscopy. **a** ^31^P MAS NMR spectra and **b** the increment of the P-O-Al bond angles of fresh and spent SAPO-34 catalysts after the MTO conversion at 623 K for different TOS of 0–3 h. **c** ^31^P and **d** ^27^Al MAS NMR spectra of spent SAPO-34 catalysts after the MTO conversion at 623 K for different TOS of 0–3 h and subsequent hydration at room temperature for 24 h.

For more information on the slight changes of the local framework structure of the SAPO-34 zeolite during the MTO conversion, the spent catalysts obtained at 623 K for different TOS were further studied by 1D ^27^Al and ^31^P MAS NMR, as well as 2D ^31^P-^27^Al HETCOR MAS NMR spectroscopy. Owing to the quadrupolar interactions of ^27^Al nuclei, similar 1D ^27^Al MAS NMR spectra of all spent SAPO-34 zeolites are found, showing the dominant broad signal at about 32.5 p.p.m. due to Al^IV^ atoms (Supplementary Fig. 11)^60^. This finding is well supported by the 2D ^31^P-^27^Al HETCOR MAS NMR spectra in Fig. 2, where a clear ^31^P-^27^Al correlation between the signals of P^IV^ and Al^IV^ atoms (≈−30.1 and 36.7 p.p.m.) occurs for all the spent catalysts. In addition, a weak ^31^P-^27^Al correlation between the P^IV^ atoms and penta-coordinated Al (Al^V^) atoms (*δ*_27Al_ ≈ 23.5 p.p.m.) occurs for the spent catalysts after TOS of 1 h. The formation of these few Al^V^ atoms could be due to the adsorption of water molecules formed by the MTO conversion at Al^IV^ atoms. With increasing TOS, the correlation signals of the above-mentioned P^IV^-O-Al^V^ species become much weaker and disappear at TOS = 3 h. This could be due to the accumulation of hydrophobic HP or coke species in the cages of the SAPO-34 catalyst (Fig. 1b), thus hindering the adsorption of water at Al^IV^ atoms, as supported by the decreasing signal of water clusters in ^1^H MAS NMR spectra (Supplementary Figs. 12 and 13). In addition, the accumulation of organic deposits in the cages of SAPO-34 leads to an obvious shift of the ^31^P MAS NMR signal from −30.1 to −30.8 p.p.m. (Fig. 3a). Interestingly, the signal shifts back after the catalyst regeneration, supporting previous arguments about the P-O-Al bond angle. Similar information can also be obtained for the spent SAPO-34 catalysts after MTO conversion at different reaction temperatures (Supplementary Fig. 14). These obvious changes determined by the ^31^P chemical shift plotted against the coke content clearly indicate that the increment of the mean P-O-Al bond angle correlates well with the amounts of organic deposits occluded in the cages of the SAPO-34 catalyst (Fig. 3b and Supplementary Fig. 15). In other words, the organic deposits can induce a reversible unit cell expansion of the SAPO-34 catalyst during the MTO conversion, which can also be well supported by in situ X-ray diffraction (XRD) results.

As shown in Supplementary Fig. 16, typical XRD patterns for the chabazite (CHA) framework structure with the similar intensities could be observed during the MTO conversion at 623 K with a TOS of 180 min (Supplementary Fig. 16a), indicating the high stability of SAPO-34 catalyst under the employed reaction conditions. Further refinement of SAPO-34 unit cell reveals that the increase in the *a*- and *c*-axis of SAPO-34 unit cell can be observed with the progress of MTO conversion (Supplementary Fig. 16b). In comparison with *a*-axis, a significant change can be observed in the *c*-axis, which is in good agreement with the previous reports of Wragg et al.^61^. These results indicate a strong link between HP formation within the cages and the expansion of the SAPO-34 unit cell, in good agreement with the aforementioned ^31^P MAS NMR results, where the increment of the mean P-O-Al bond angle correlates well with the amounts of organic deposits occluded in the cages of the SAPO-34 catalyst. These results can also be well supported by density functional theory (DFT) results, where an obvious increase of the mean distance of P-Al bond in SAPO-34 unit cell occurs upon the formation of aromatics within the cages (Supplementary Fig. 16c).

**Fig. 4:**
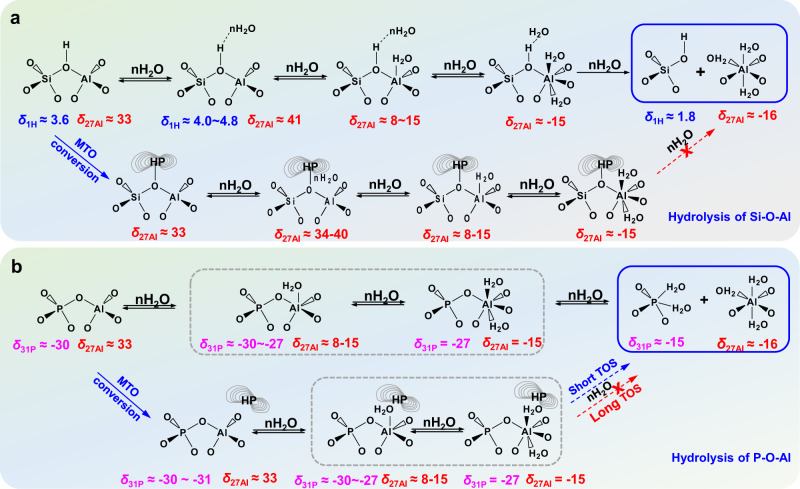
Proposed hydrolysis process of T-O-T bonds. Schematic illustration of the hydrolysis process of framework Si-O-Al (**a**) and P-O-Al (**b**) bonds for fresh and spent SAPO-34 catalysts after the MTO conversion.

Previous studies indicated that the SAPO-34 zeolite is very sensitive to moisture and hydrolysis of the P-O-Al bonds occurs already at room temperature^55^. For studying the water-induced dynamic structure changes of the fresh SAPO-34 sample, ^27^Al and ^31^P MAS NMR spectra of this catalyst after hydration for up to 1 week were recorded (Supplementary Fig. S17). Upon the hydration of the spent SAPO-34 catalyst, new ^27^Al NMR signals occur at about 15.3 and −15.7 p.p.m., due to Al^V^ and Al^VI^ atoms coordinated to one and two water molecules^62^, respectively (Supplementary Fig. 9b). Simultaneously, the ^31^P MAS NMR signals of the P^IV^ atoms (−30.1 ~ −30.3 p.p.m.) shift to −28.5 ~ −29.7 p.p.m. and a weak shoulder at about −15.4 p.p.m. appears (Supplementary Fig. 10). According to a previous study^56^, the latter ^31^P MAS NMR signal should be due to penta-coordinated P (P^V^) atoms caused by the hydrolysis of P-O- Al bonds (as shown in Fig. 4). In comparison with the fresh and regenerated SAPO-34 samples, the intensities of the aforementioned ^27^Al MAS NMR signals at 15.3 and −15.7 p.p.m., and the ^31^P MAS NMR signals at −15.4 p.p.m. decreased dramatically. It demonstrates that hydration of the spent SAPO-34 catalysts was hindered by the presence of organic deposits.

To demonstrate the water-induced dynamic structure changes of spent SAPO-34 catalysts after the MTO conversion, 1D ^27^Al and ^31^P MAS NMR, as well as 2D ^31^P-^27^Al HETCOR MAS NMR spectra of the hydrated SAPO-34 catalysts obtained after the MTO conversion with different TOS are also shown in Fig. 3c, d and Supplementary Figs. 18–20. After hydration, broad ^31^P MAS NMR signals at about −28.4 p.p.m. with a small shoulder at −15.4 p.p.m. occur in the spectra of the spent catalysts. With increasing TOS, the ^31^P MAS NMR signal at −28.4 p.p.m. shifts to the high-field direction (from −28.4 to −30.4 p.p.m.) and becomes more narrow (Fig. 3c). Simultaneously, the ^31^P MAS NMR signals at −15.4 p.p.m. gradually decreases and disappears at TOS = 3 h. Analogously, the ^27^Al MAS NMR signals at 15.4 and −15.7. p.p.m. of the respective hydrated catalysts gradually decrease with increasing TOS (Fig. 3d). This is in accordance with the 2D ^31^P-^27^Al HETCOR MAS NMR spectra, where at least four ^31^P-^27^Al correlations between the P^IV^ and Al^IV^ atoms occur for the spent catalysts obtained with the short TOS of 20 min (Supplementary Fig. 20). With increasing TOS, the ^31^P-^27^Al correlations between the P^IV^ and Al^IV^ atoms gradually decrease and only one correlation (−30.7 and 36.7 p.p.m.) is left after TOS = 2 h. Apart from the aforementioned correlations, no correlation between P^IV^ and Al^V^ or P^IV^ and Al^VI^ atoms (Fig. 4b, marked with dotted line) is observed in the spectrum, revealing that the 1D ^27^Al MAS NMR signal at 15.4 p.p.m. should be due to Al^V^ atoms in the local structure of bridging OH groups (Si-OH-Al), but not from P-O-Al bonds. Because if the ^27^Al MAS NMR signal at 15.4 p.p.m. originated from P-O-Al bonds, there will be an obvious correlation between P^IV^ and Al^V^ in 2D ^31^P-^27^Al HETCOR MAS NMR spectra, while it is absent. The ^27^Al MAS NMR signal at −15.7 p.p.m., on the other hand, should be due to Al^VI^ atoms in bridging OH groups interacting with two water molecules or caused by the hydrolysis of P-O-Al bonds (Fig. 4). The hydrolysis of Si-O-Al bonds in the spent SAPO-34 catalysts obtained with short TOS can be excluded, as Si-OH signals at 1.8 p.p.m. are absent in the ^1^H MAS NMR spectra (Supplementary Fig. 12). In contrast, the ^31^P MAS NMR signal at −15.4 p.p.m. should be due to the hydrolysis of P-O-Al bonds. Hence, for the spent SAPO-34 catalysts after short TOS, a hydrolysis of P-O-Al bonds occurs, but not of Si-O-Al bonds. With progress of the MTO conversion, more organic deposits are occluded in the pores and cages of the SAPO-34 catalysts, which partially cover BAS and even some P-O-Al bonds, thus preventing the framework hydrolysis. Namely, the organic deposits in the pores of SAPO-34 catalysts can effectively protect them against framework hydrolysis.

In summary, the HP and coke compounds formed during the MTO conversion can lead to a reversible unit cell expansion of SAPO-34 catalysts, thus causing a gradual increase of the mean P-O-Al bond angles with the progress of the MTO conversion. In addition, the organic deposits occluded in the pores and cages of SAPO-34 catalysts can protect the framework against hydrolysis, thus improving their stability.

### Improvement of the long-term stability of SAPO-34 catalysts

**Fig. 5:**
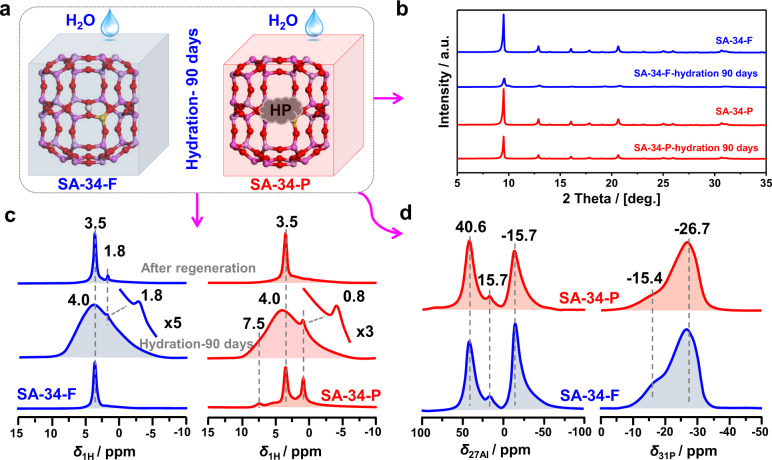
Stability of SAPO-34 zeolite after long-term hydration. **a** Schematic diagrams of the hydration strategy for fresh and pre-coked SAPO-34 zeolites at room temperature for 90 days. **b** XRD patterns of the fresh and pre-coked SAPO-34 samples before and after hydration for 90 days. **c** ^1^H MAS NMR spectra of the dehydrated, hydrated, and regenerated SAPO-34 samples before and after pre-coking. **d** ^27^Al and ^31^P MAS NMR spectra of the fresh and pre-coked SAPO-34 samples after hydration for 90 days at room temperature.

Previous studies indicated that the calcined SAPO-34 zeolites are very sensitive to moisture and complete hydrolysis could happen after longtime storage at room temperature^27^. The aforementioned spectroscopic results clearly indicate that the organic deposits in the cages of SAPO-34 catalyst can efficiently prevent the hydrolysis of T-O-T bonds. Therefore, the pre-coking of SAPO-34 catalysts is expected to be an efficient strategy to improve their long-term stability in the presence of moisture. For comparison, SAPO-34 catalysts before and after pre-coking were exposed to moisture for 90 days, to evaluate their long-term stability (Fig. 5a). As shown in Fig. 5b, only a slight decrease (45%) in the intensity of the XRD patterns can be observed for the pre-coked SAPO-34 catalyst (SA-34-P), indicating that its CHA structure can be well preserved after longtime hydration. In contrast, an obvious decrease (84%) in the XRD patterns with partial framework collapse occurs for the fresh SAPO-34 sample (SA-34-F). This can be well supported by the ^1^H MAS NMR spectra, where a weak signal at 1.8 p.p.m. due to Si-OH groups occurs for the hydrated SA-34-F sample. According to the previous studies^23,55^, the Si-OH groups could be due to the hydrolysis of Si-O-Al or Si-O-Si bonds, whereas the Si-O-Si bonds in SAPO-34 zeolite under study can be excluded by ^29^Si MAS NMR spectra (Supplementary Fig. 21), where no Si islands occur in the range of −100 ~ −110 p.p.m.^22^. Therefore, the ^1^H MAS NMR signal at 1.8 p.p.m. under study should be due to the Si-OH groups, owing to the hydrolysis of Si-O-Al bonds (Fig. 5c). After regeneration, the Si-OH signal at 1.8 p.p.m. can be still observed, revealing that the hydrolysis of Si-O-Al bonds is irreversible. On the contrary, the Si-OH groups are absent in the SA-34-P catalyst, clearly illustrating that the pre-coking is an effective strategy to enhance the stability of SAPO-34 zeolites. Furthermore, in comparison with SA-34-P, obvious increases of the ^27^Al MAS NMR signal at −15.7 p.p.m. and the ^31^P MAS NMR signal at −15.4 p.p.m. occur for the SA-34-F sample after longtime hydration, also revealing that a severe hydrolysis of T-O-T bonds occurred in the SA-34-F sample (Fig. 5d). Overall, the pre-coking of SAPO-34 catalysts can efficiently protect their framework against hydrolysis of T-O-T bonds, thus enhancing their long-term stability in the presence of moisture at room temperature.

**Fig. 6:**
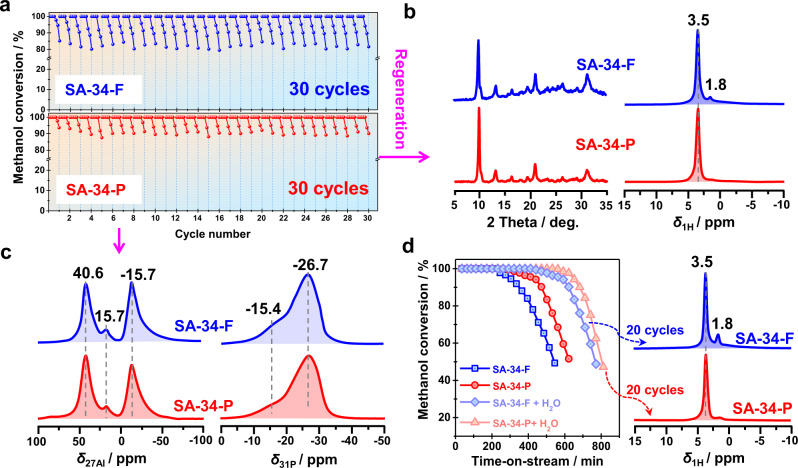
Stability of SAPO-34 zeolite after continuous repeated regenerations. **a** Recycling studies of SAPO-34 catalysts applied in the MTO conversion at 673 K with and without pre-coking. **b** XRD patterns and ^1^H MAS NMR spectra of the regenerated SAPO-34 catalysts after 30 cycles in the MTO conversion. **c** ^27^Al and ^31^P MAS NMR spectra of the regenerated SAPO-34 catalysts after 30 cycles in the MTO conversion and after hydration at room temperature for 24 h. **d** Methanol conversion before and after water co-feeding (20 Vol%) over SA-34-F and SA-34-P catalysts at 673 K (left). ^1^H MAS NMR spectra of the regenerated SAPO-34 catalysts after 20 cycles in the MTO conversion with water co-feeding (right).

A recent report indicates that water molecules formed during the MTO conversion compete with alkenes for adsorption at the BAS under reaction conditions^38^. Therefore, the effect of water formed by the MTO conversion over SAPO-34 catalysts was further studied. As shown in Fig. 6a, the MTO activities of SA-34-F and SA-34-P catalysts upon 30 cycles were compared (the experimental details are given in the “Methods” section), and a comparable activity was observed for these two catalysts. After 30 cycles, nearly no activity loss occurred for these two catalysts in comparison with the fresh sample, but a slight difference in the framework structure appeared. In comparison with the SA-34-P catalyst, an obvious decrease in the XRD patterns occurred for SA-34-F, indicating the occurrence of a partial framework collapse upon 30 cycles. This was also supported by ^1^H MAS NMR spectroscopy, as a signal of Si-OH groups (*δ*_1H_ = 1.8 p.p.m.), owing to the hydrolysis of Si-O-Al bonds, appeared for the SA-34-F catalyst, but can be negligible for the SA-34-P catalyst (Fig. 6b). This finding is also in accordance with the ^27^Al and ^31^P MAS NMR spectra of the SA-34-F catalyst, which showed an increase of the ^27^Al MAS NMR signal at −15.7 p.p.m. and of the ^31^P MAS NMR signal at −15.4 p.p.m. due to the hydrolysis of T-O-T bonds (Fig. 6c). These results indicate that water could also lead to a partially irreversible hydrolysis of Si-O-Al bonds during the long-term running of the MTO conversion, thus causing a partial framework collapse. After pre-coking, however, the hydrolysis of Si-O-Al bonds can be effectively prevented, thus a long-term stability of SAPO-34 catalyst would be expected in the commercial MTO process.

The stability of pre-coked SAPO-34 catalyst in the high-temperature steaming procedures was also evaluated. As shown in Supplementary Fig. 22, only a slight decrease (10%) in the intensity of the XRD patterns can be observed for the fresh sample after steaming at 973 K for 30 h, indicating its good hydrothermal stability, in line with the previous studies of Corma et al.^22^. After pre-coking, however, no obvious changes in the XRD patterns occur, hinting the stability of SAPO-34 material can be improved by the presence of organic deposits. This can be well supported by the ^27^Al and ^31^P MAS NMR spectra, where obvious decrease of the ^27^Al MAS NMR signal at −15.7 p.p.m. and the ^31^P MAS NMR signal at −15.4 p.p.m. occur for the SA-34-P sample after steaming (Supplementary Figs. 22b, c and  23), revealing that pre-coking of SAPO-34 catalyst can effectively protect the framework against the hydrolysis of T-O-T bonds in the high-temperature steaming. In addition, the same shift of the ^31^P MAS NMR signal from −30.1 to −30.8 p.p.m. can also be observed over the pre-steamed SAPO-34 catalysts after MTO conversion at 623 K with different TOS of 0–3 h (Supplementary Fig. 24). These results indicate that the high-temperature steaming has no impact on the ^31^P MAS NMR results also confirm the reproducibility and repeatability of this method.

Water co-feeding is frequently utilized in the MTO conversion to increase the olefin selectivity and to prolong the catalyst single-pass lifetime. Therefore, the long-term stability of SAPO-34 catalysts during the MTO conversion after water co-feeding was also evaluated for comparison. As shown in Fig. 6d, the catalyst single-pass lifetime can be significantly increased after co-feeding 20 Vol% water, revealing that the coking rate can be greatly reduced, in accordance with previous reports^38,40,41^. However, after 20 cycles, a slight decrease in the MTO activity occurs for SA-34-F catalyst and the catalyst single-pass lifetime decreases from the initial 400 min to about 300 min (Supplementary Fig. 25a). Furthermore, an obvious ^1^H MAS NMR signal at 1.8 p.p.m. occurs in the spectrum of the regenerated catalyst after 20 cycles, indicating water co-feeding can accelerate the hydrolysis of Si-O-Al bonds, as, especially in the initial phase, the catalyst is freely exposed to water. In contrast, no obvious decline in the catalytic performance (Supplementary Fig. 25b) and only a very weak ^1^H MAS NMR signal at 1.8 p.p.m. occurred for the SA-34-P catalyst (Fig. 6d). This result clearly indicates that water co-feeding in the MTO conversion can distinctly prolong the catalyst single-pass lifetime, but also cause a partial damage of the catalyst framework upon long-term recycles. However, the pre-coking of SAPO-34 catalyst can effectively protect the framework against an irreversible hydrolysis of Si-O-Al bonds in the initial reaction phase.

In a commercial MTO process, pre-coking of fresh catalyst or partial regeneration of deactivated catalyst is usually employed to shorten the induction period and enhance the initial product selectivity^1,63–65^. Our findings on the pre-coking-induced steady framework of SAPO-34 zeolite during the long-term MTO conversion in the present study provide new understanding of the dynamic changes of the SAPO-34 framework during the MTO conversion and shed light on the development of other commercial zeolite catalysts with high stability.

## Discussion

In summary, subtle changes of the local framework structure of SAPO-34 catalysts during the MTO conversion have been identified via 1D ^27^Al and ^31^P MAS NMR, as well as 2D ^31^P-^27^Al HETCOR MAS NMR spectroscopy. The HP species and coke compounds formed during the MTO conversion can lead to a reversible unit cell expansion of SAPO-34 catalysts, thus causing a gradual increase of the mean P-O-Al bond angle with progress of the MTO conversion. In the presence of water, a reversible hydrolysis of P-O-Al bonds without damaging the catalyst framework can occur rapidly for SAPO-34 catalysts. However, after longtime exposure to moisture or continuously repeated regenerations in the MTO conversion, the irreversible hydrolysis of Si-O-Al bonds in SAPO-34 catalyst with partial framework degradation would occur. After water co-feeding, the catalyst single-pass lifetime in the MTO conversion could be greatly prolonged, but the irreversible hydrolysis of Si-O-Al bonds, causing the partial framework collapse, happens likewise after 20 regeneration cycles. Interestingly, the aforementioned hydrolysis of T-O-T bonds can be strongly prevented by catalyst pre-coking. Consequently, the combination of catalyst pre-coking and water co-feeding is established to be an efficient strategy to promote the catalytic efficiency and the long-term stability of SAPO-34 catalysts in the commercial MTO processes. This fundamental work also sheds light on the development of other highly stable zeolite catalyst in the commercial catalysis.

## Methods

### Materials and physicochemical characterization

The SAPO-34 catalyst utilized in the present work was obtained from the Catalyst Plant of Nankai University (Tianjin, China). The physicochemical properties of the calcined SAPO-34 catalyst are given in Supplementary Table S1. XRD pattern of the calcined sample was recorded on a Rigaku Smart Lab 3 kW with Cu Kα radiation (*λ* = 1.5418 Å, 40 mA, 40 kV) with a scan rate of 6°/min. The particle size and the morphology of the fresh SAPO-34 catalyst was investigated on the JEOL-JSM7500 field-emission scanning electron microscope (SEM) and a thin layer of gold sputtering covering the sample was conducted before the SEM measuring. The chemical compositions of the calcined SAPO-34 catalyst were measured by inductively coupled plasma atomic emission spectrum (IRIS Advantage). The BET surface area and the microporous volume of the fresh and used SAPO-34 catalysts were measured by argon adsorption–desorption methods at low reaction temperature (87 K) on a Quantachrome iQ-MP instrument. Before argon adsorption, the samples were pretreated at 393 K for 6 h under vacuum condition to remove the water molecules. The total surface area was calculated via the BET equation and the microporous pore volume was determined using the *t*-plot method.

### Catalytic evaluation

The MTO reaction was performed in a fixed-bed quartz tubular reactor with an inner diameter of 8 mm under atmospheric pressure. First, the SAPO-34 catalyst was pressed into tablets under the pressure of 20 Mpa for 10 min and then was sieved to obtain aggregate particle sizes between 200 and 315 μm. Prior to contacting with the reactants, 0.2 g sample was heated to 723 K using nitrogen as the carrier gas with the flow rate of 25 ml/min for 1 h and then cooled to the desired temperature in a continuous flow of nitrogen. Thereafter, methanol was fed into the fixed-bed by the carrier gas of nitrogen corresponding to a weight hourly space velocity of 1.0/h. The reaction products were analyzed by an on-line gas chromatograph equipped with a flame ionization detector and a Plot Q capillary column to separate the reaction products. The following temperature program was employed: isothermal heating at 323 K for 3 min, heating to 423 K with a rate of 15 K/min, and isothermal heating at 423 K for 3 min, further heating to 473 K with a rate of 7 K/min, and isothermal heating at 473 K for 5 min. In this work, dimethyl ether in the effluent was considered as the reactant.

For preparing the pre-coked SAPO-34 catalyst, 0.2 g of the catalyst (sieve fraction, 200–315 μm) was placed in a quartz tubular reactor and activated under flowing nitrogen gas at 723 K for 1 h. After the temperature was decreased to 673 K, ethene diluted with high-purity helium was introduced and maintained for 5 min. The catalyst after ethene conversion for 5 min was named as pre-coked SAPO-34 (SA-34-P). Thereafter, methanol was fed into the fix bed with the pre-coked materials for further MTO conversion.

For regenerating the SA-34-F catalysts, the carrier gas was changed as synthetic air after MTO conversion and the deactived catalysts were treated in the synthetic air at 773 K for 6 h in the fixed-bed reactor. After regeneration, the synthetic air was changed back into nitrogen and, thereafter, the new MTO cycle was started. For SA-34-P regeneration, only ethene diluted with high-purity helium was introduced and maintained for 5 min before the new MTO cycle and other processes are the same as that of SA-34-F catalysts.

### Characterization of carbonaceous species

The nature of the organic species formed on the SAPO-34 catalyst during the MTO conversion were investigated by in situ UV-vis spectroscopy. The UV-vis spectra were recorded in the diffuse reflection mode in the range of 200–600 nm using an AvaSpec-2048 fiber optic spectrometer, an AvaLight-DH-S deuterium light source by Avantes, and a glass fiber reflection probe HPSUV1000A by Oxford Electronics. Before starting the MTO reaction, the glass fiber reflection probe was placed in the fixed-bed reactor on the top of the catalyst with a gap of ∼1.0 mm to the catalyst bed. Reference UV-vis spectra of the catalysts were recorded at the desired reaction temperature prior to starting the methanol flow and the reaction conditions were the same as those described in “Catalytic evaluation” section. The amount of the organic deposits occluded in the cages of SAPO-34 catalysts after MTO conversion was analyzed by thermogravimetric analysis on the SDT Q600 thermogravimetric analyzer. Typically, 5 mg of the used catalyst was heated in an Al_2_O_3_ crucible from 298 to 1073 K with a heating rate of 10 K/min under the air flow of 100 ml/min. The nature of occluded hydrocarbons in the catalysts after MTO conversion with different TOS was analyzed by gas chromatography–mass spectrometry (GC-MS). Typically, 0.1 g of the spent catalyst was carefully dissolved in 1 M hydrofluoric acid (HF) solution, which was then treated with CH_2_Cl_2_ to extract the organic compounds and the residual water was removed by the addition of sufficient anhydrous sodium sulfate solid. Then, 0.2 μL of the organic extract was analyzed by GC-MS (GC-MS-QP2010 SE) with a RXI-5MS column (30 m, 0.25 mm i.d., stationary phase thickness 0.25 μm). The following temperature program was employed: isothermal heating at 313 K for 3 min, heating to 553 K with a rate of 10 K/min, and isothermal heating at 553 K for 8 min. In situ XRD patterns were determined by Bruker D8 with Cu *K*_α_ radiation (*λ* = 1.5418 Å) equipped with an in situ reaction chamber. Prior to the XRD studies, the SAPO-34 catalysts were finely ground and placed in the chamber. Then, the catalysts were activated in flowing argon gas at 673 K for 1 h and, subsequently, decreased to 623 K for MTO conversion. Thereafter, the XRD patterns were collected with a scan rate of 3°/min. XRD patterns were analyzed by using the software TOPAS 5.0. Unit cells were refined by using unit cell parameters of the ideal **CHA** framework as an initial model, which was downloaded from International Zeolite Association. The space group of idealized **CHA** structure is *R*-3*m* (No. 166), where there are one T atom and four O atoms in the asymmetric unit. However, due to the alternating distribution of Al and P in SAPO-34, the space group would be reduced to *R*-3 (No. 148).

### Structured illumination microscopy

SIM was applied to gain super-resolution images on the spatiotemporal distribution of the coke species located in the spent SAPO-34 samples after MTO conversion with different TOS. The super-resolution imaging was carried out using a Nikon N-SIM super-resolution microscopy system with a motorized inverted microscopy ECLIPSE Ti2-E, a ×100/numerical aperture 1.49 oil-immersion total internal reflection fluorescence objective lens (CFI HP) and ORCA-Flash 4.0 sCMOS camera (Hamamatsu Photonics K.K.)^66,67^. Imaging with 405, 488, 561, and 640 nm multilaser light sources, the emissions by excitation were collected with four photomultiplier tubes in the range 435–485, 500–545, 570–640, and 663–738 nm for the four lasers, respectively. Imaging was performed under ex situ condition at room temperature. The spent SAPO-34 samples were loaded on glass-bottomed culture dishes (35 mm dish with 20 mm well) and images were taken at a *Z*-plane of middle of zeolitic crystal. The software NIS-Elements Ar and N-SIM Analysis were used to analyze the collected images and computationally reconstruct the super-resolution image.

### Solid-state NMR measurements

Solid-state NMR studies of the fresh and spent SAPO-34 samples in the hydrated and dehydrated states were performed on a Bruker Avance III 400WB spectrometer at resonance frequencies of 400.1, 104.3, and 161.9 MHz for ^1^H, ^27^Al, and ^31^P nuclei, respectively. The experimental conditions were as follows: single pulse excitation of *π*/2 for ^1^H and ^31^P, *π*/6 for ^27^Al, and repetition times of 20 s for ^1^H, 0.5 s for ^27^Al, and 35 s for ^31^P MAS NMR spectroscopy. The ^1^H, ^27^Al, and ^31^P MAS NMR spectra were recorded with a sample spinning rate of 8 kHz using a 4 mm MAS NMR probe. The hydrated samples after different hydration times were obtained by exposing them to an atmosphere saturated with the vapor of an aqueous solution of Ca(NO_3_)_2_ at room temperature. The ^27^Al chemical shifts were also referenced to 1 M Al(NO_3_)_3_ aqueous solution (0 p.p.m.), whereas the ^31^P and ^1^H chemical shifts were referenced to NH_4_H_2_PO_4_ (0.8 p.p.m.) and adamantane (1.74 p.p.m.), respectively.

The acidity of the fresh and spent SAPO-34 samples obtained under different reaction conditions was characterized by ^1^H MAS NMR spectroscopy. Before the ^1^H MAS NMR studies of the fresh SAPO-34 samples, these materials were dehydrated at 673 K in vacuum (pressure below 10^−2^ Pa) for 10 h. Subsequently, the materials were sealed and kept in glass tubes until their transfer into gas-tight MAS NMR rotors inside a glove box purged with dry nitrogen gas. The used SAPO-34 catalysts were obtained after quenching the MTO conversion and transferring them into MAS NMR rotors without contact to air. The determination of the number of accessible BASs was performed upon adsorption of ammonia at room temperature and by evaluating the ^1^H MAS NMR signals caused by ammonium ions (*δ*_1H_ = 6.0–7.0 p.p.m.). For this purpose, the catalyst samples were loaded with 100 mbar ammonia and, subsequently, evacuated at 453 K for 2 h to eliminate physisorbed ammonia. Quantitative ^1^H MAS NMR measurements were performed by comparing the signal intensities of the samples under study with the intensity of an external intensity standard (dehydrated zeolite H,Na-Y with the cation exchange degree of 35%). The decomposition and simulation of NMR spectra were carried out utilizing the Bruker software WINFIT. Moreover, the amounts of adsorbed water on the catalysts were also obtained via a similar method.

The 2D ^31^P-^27^Al HETCOR MAS NMR spectra were recorded at 14.1 T on a Varian 600 spectrometer using commercial 4 mm rotors at a spinning rate of 10 kHz, tuned and matched at the ^1^H, ^31^P, and ^27^Al Larmor frequencies (599.81, 242.80, and 156.29 MHz, respectively). For the 2D ^31^P-^27^Al HETCOR MAS NMR experiments, the Hartmann–Hahn condition was achieved using our own samples, with a cross polarization (CP) contact time of 2.2 ms and a repetition time of 1 s. The number of scans and t1 increments were set to 400 and 40, respectively. The ^31^P chemical shifts were referenced to diammonium phosphate (1 p.p.m.). The ^27^Al chemical shifts were referenced to 1 M Al(NO_3_)_3_ aqueous solution (0 p.p.m.).

### Calculation method

SAPO-34 zeolite is represented by a unit cell, which was extracted from CHA crystallographic structural data (http://www.iza-structure.org/databases/). The geometry optimizations were performed by using the DMol3 program^68,69^ with the double numerical polarization basis set and the generalized gradient-corrected Perdew–Burke–Ernzerhof functional. The default medium level Monkhorst-Pack *K*-point (1 × 1 × 1) was adopted to sample the Brillouin zone. The convergence criteria for energy, force, and displacement were 2 × 10^−5^ hartree, 4 × 10^−3^ hartree/Å, and 5 × 10^−3^ Å, respectively. Dispersion corrected density functional theory (DFT-D) method was used in the structure optimization to accurately describe the weak interaction between SAPO-34 zeolite and the hydrocarbon species^70^. During optimization, all of framework atoms and the adsorbed the hydrocarbon species were relaxing to their equilibrium positions.

## Supplementary information

Supplementary Information

## Data Availability

All data presented in this paper are available from the corresponding authors upon request.
