# Supplementary material for: Stabilizing the framework of SAPO-34 zeolite toward long-term methanol-to-olefins conversion
Source: Nat Commun. 2021 Aug 2;12:4661. doi: 10.1038/s41467-021-24403-2 (PMC8329068; doi:10.1038/s41467-021-24403-2)
Supplement: Supplementary file 1 — Supplementary Information [file 41467_2021_24403_MOESM1_ESM.pdf]

Supplementary Information

Stabilizing the Framework of SAPO-34 Zeolite toward Long-term Stable Methanol-to-olefins Conversion

Yang *et al.*

Supplementary Figures

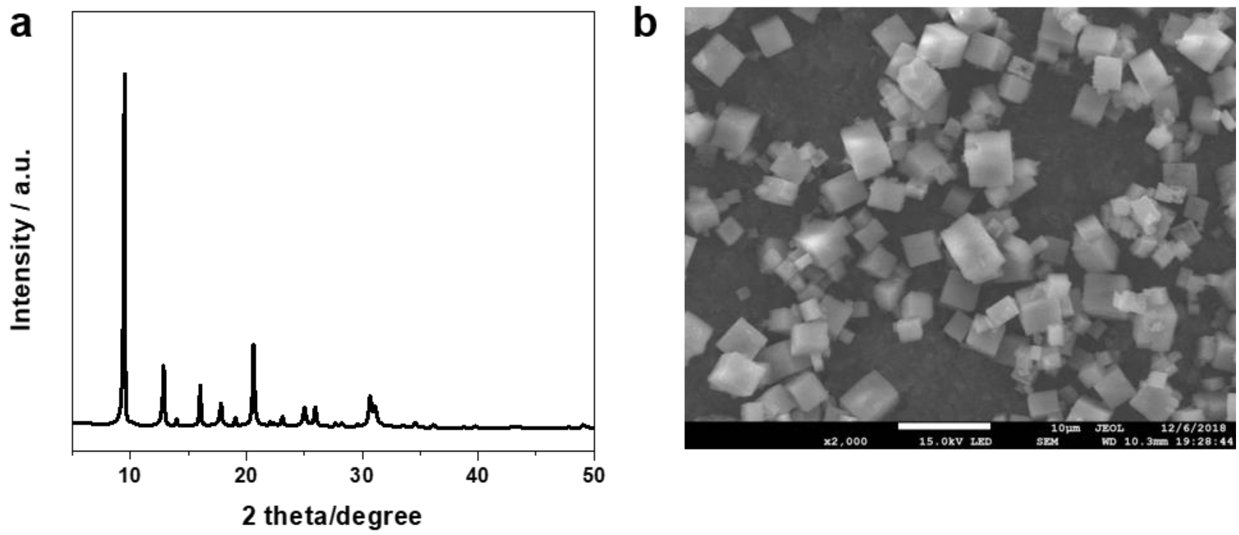

Supplementary Fig. 1 XRD patterns of the calcined SAPO-34 sample (a) and SEM picture of the as-synthesized SAPO-34 sample under study (b).

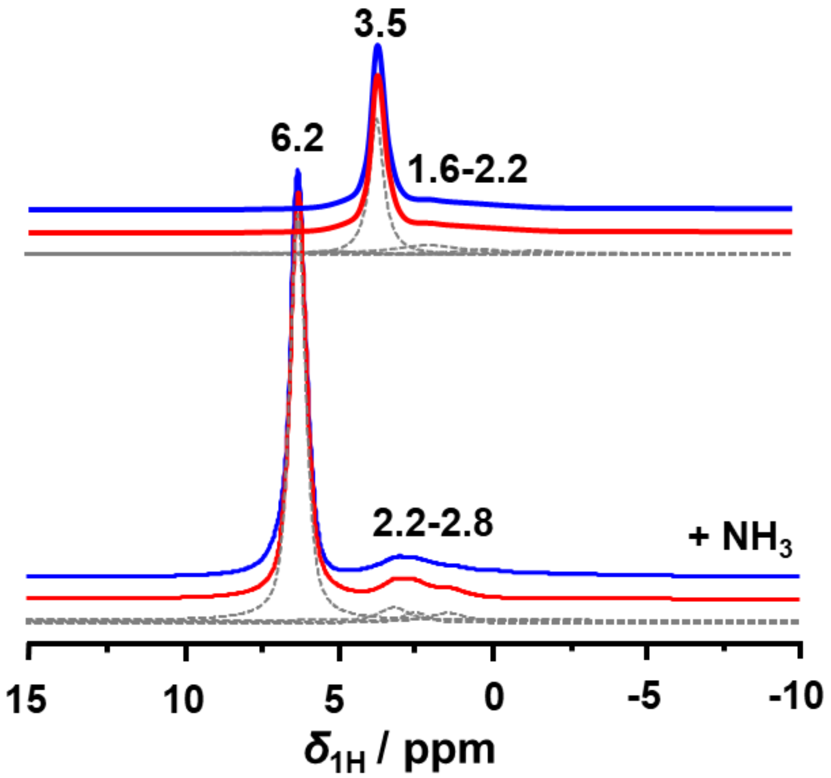

Supplementary Fig. 2 ^1H MAS NMR spectra of the fresh SAPO-34 sample, recorded before and after adsorption of ammonia. From top to bottom, the experimental spectra, the simulated spectra, and the signal components utilized for the simulation are shown.

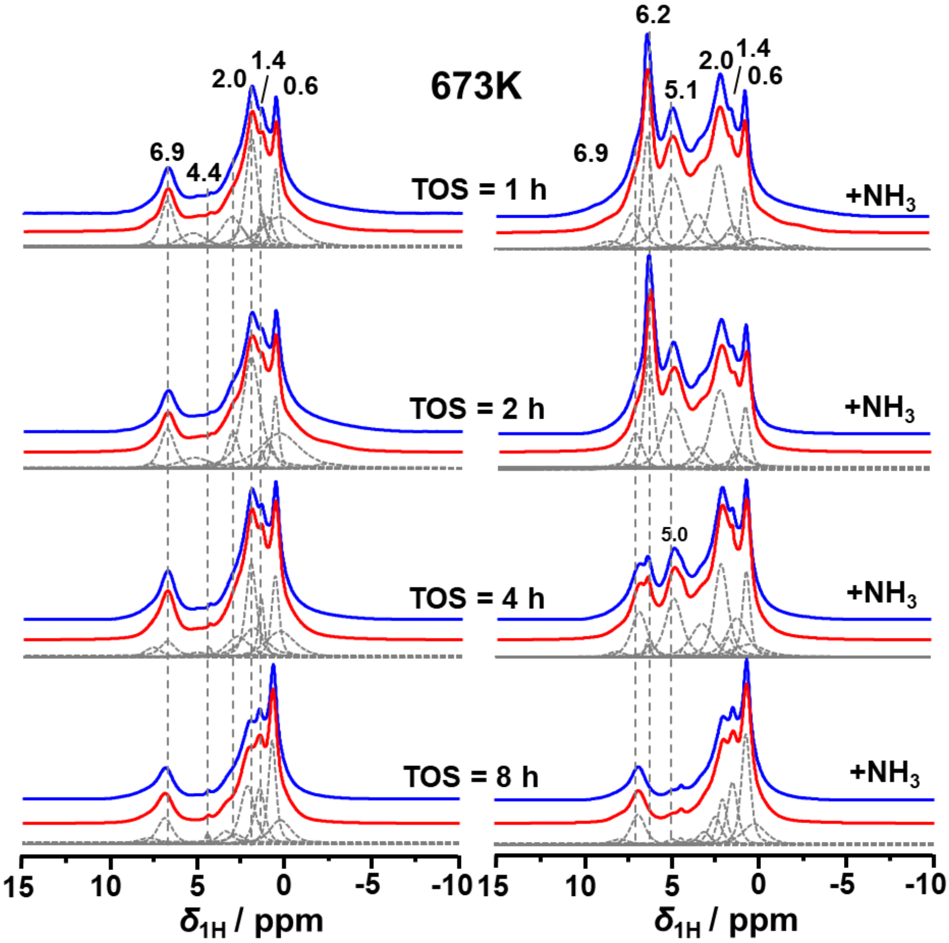

Supplementary Fig. 3 ^{1}H MAS NMR spectra of the spent SAPO-34 catalysts obtained after MTO conversion at 623 K with different TOS.

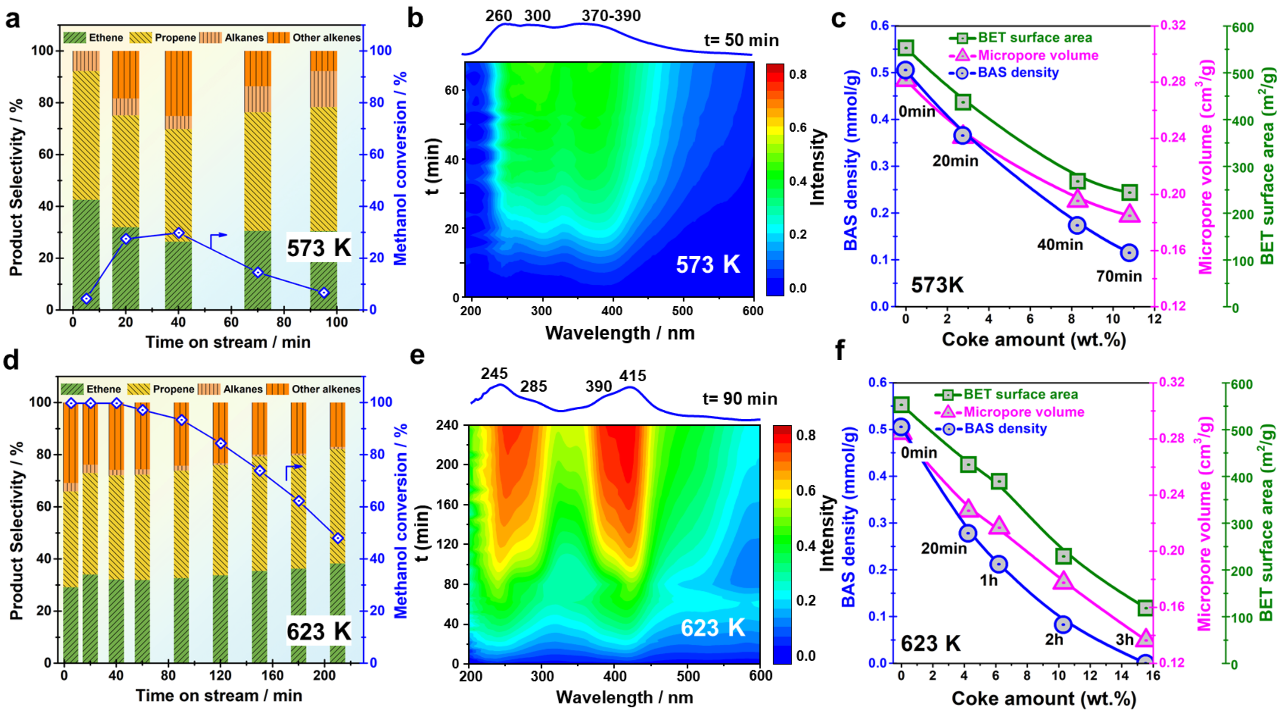

Supplementary Fig. 4 Methanol conversion and product distribution during the MTO conversion over SAPO-34 catalyst at 573 (a) and 623 K (d). *In situ* UV-vis spectra of MTO conversion over SAPO-34 recorded at 573 (b) and 623K (e). In c and f, the number of accessible Brønsted acid sites, the BET surface area and micropore volume of the SAPO-34 catalyst during the MTO conversion at 573 and 623 K are plotted as a function of coke amounts.

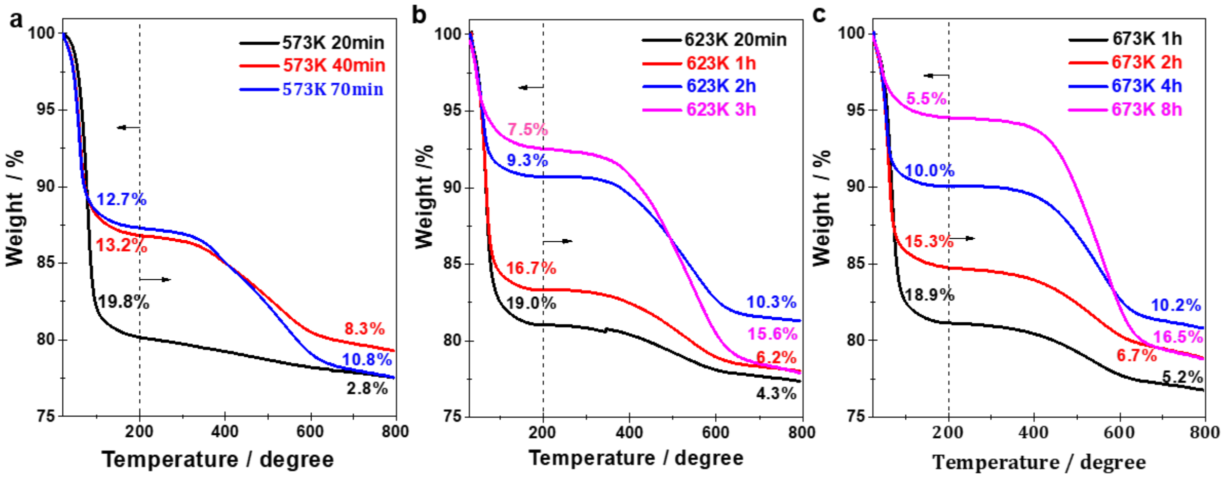

Supplementary Fig. 5 TGA curves of the spent SAPO-34 catalysts obtained after MTO conversion at 573 (a), 623 (b) and 673K (c) with different TOS.

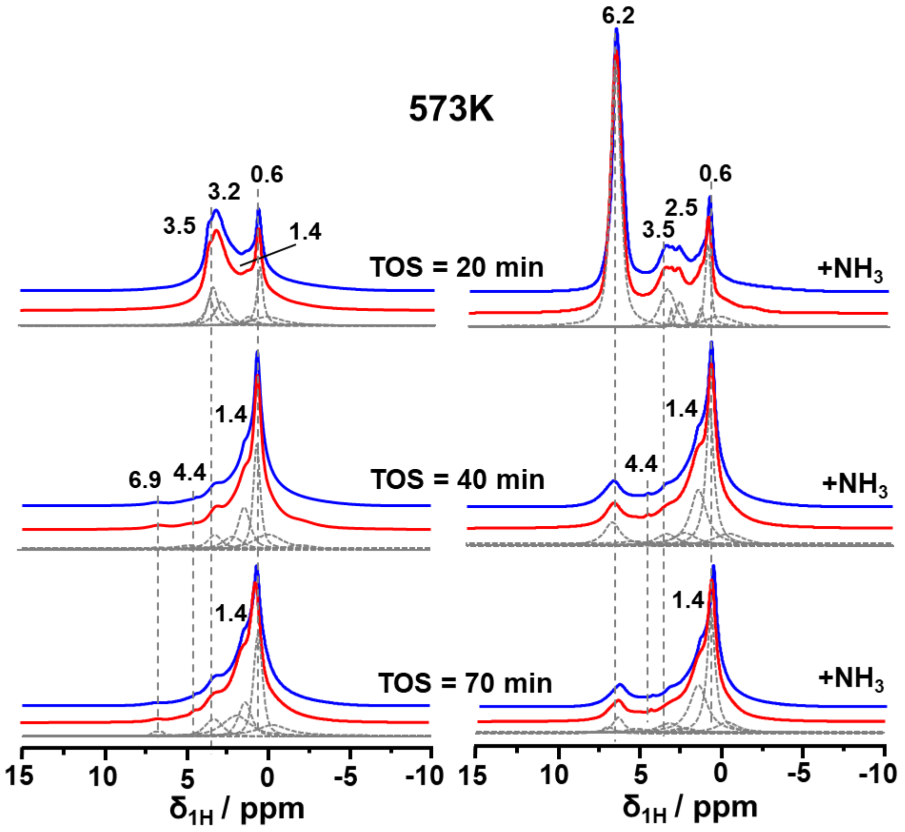

Supplementary Fig. 6 ^{1}H MAS NMR spectra of the spent SAPO-34 catalysts obtained after MTO conversion at 573 K with different TOS.

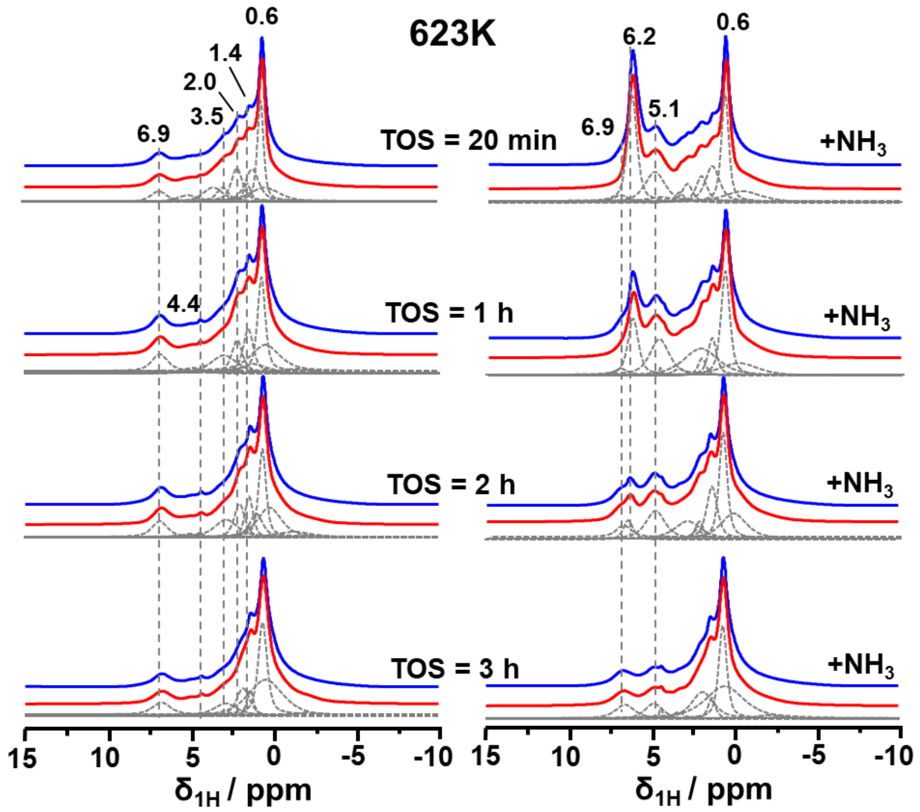

Supplementary Fig. 7 ^{1}H MAS NMR spectra of the spent SAPO-34 catalysts obtained after MTO conversion at 623 K with different TOS.

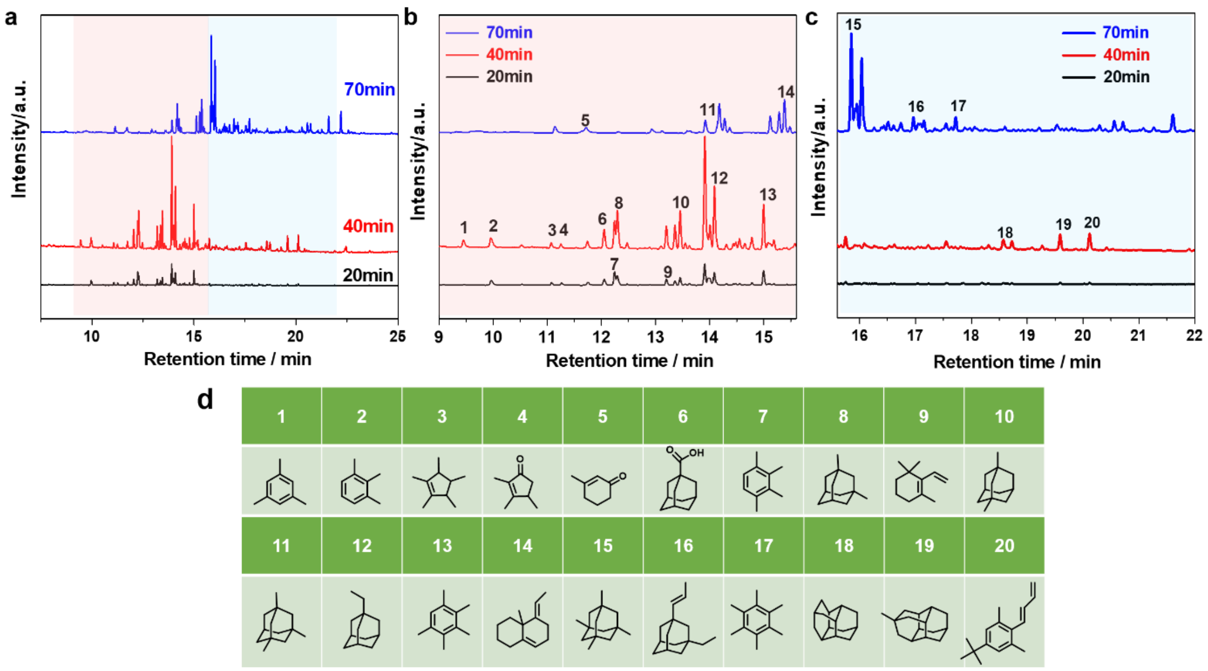

Supplementary Fig. 8 GC-MS chromatograms (a, b and c) and the structures (d) of the organic extracts from spent SAPO-34 catalysts obtained after MTO conversion at 623 K with different TOS.

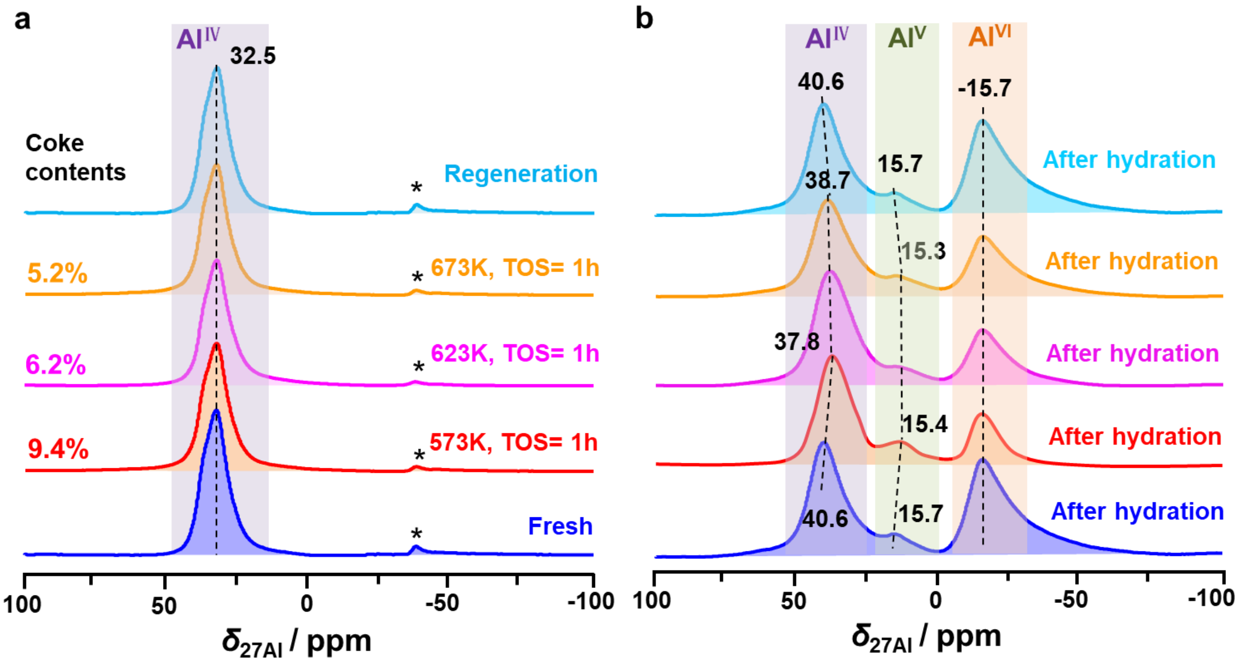

Supplementary Fig. 9 ^{27}Al MAS NMR spectra of the spent SAPO-34 catalysts obtained after MTO conversion at different temperatures with different TOS, recorded before (a) and after (b) hydration at room temperature for 24 h.

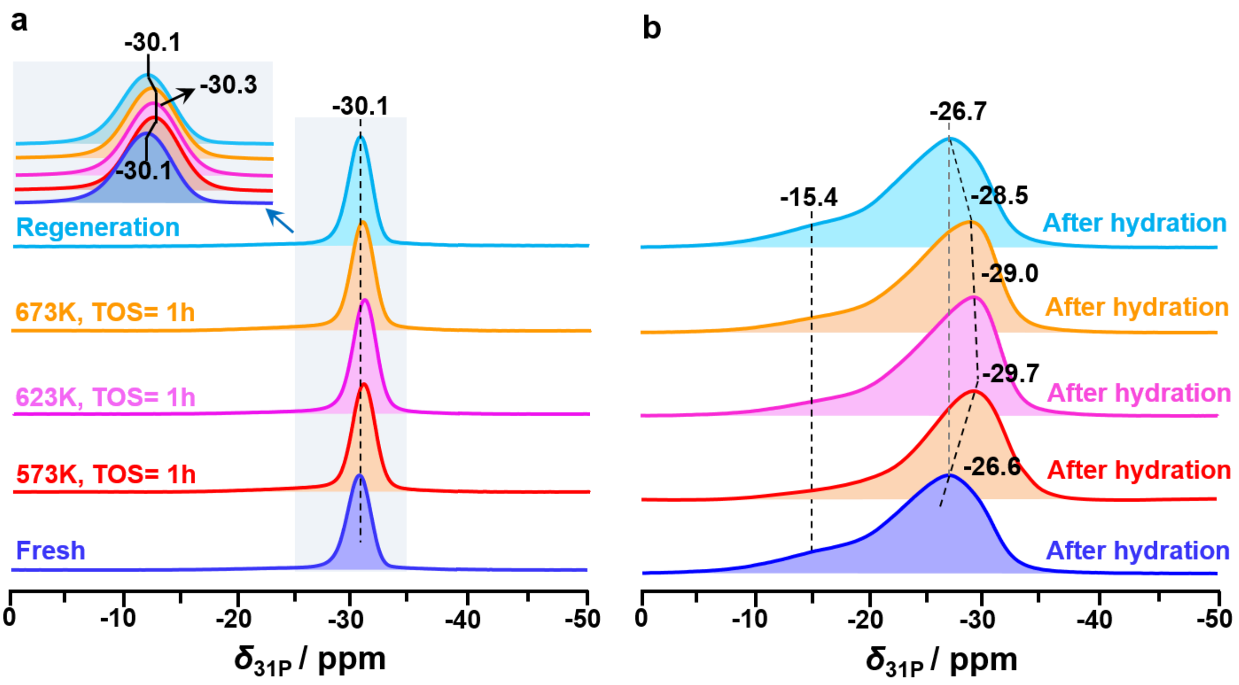

Supplementary Fig. 10 ^{31}P MAS NMR spectra of the spent SAPO-34 catalysts obtained after MTO conversion at different temperatures with different TOS, recorded before (a) and after (b) hydration at room temperature for 24 h.

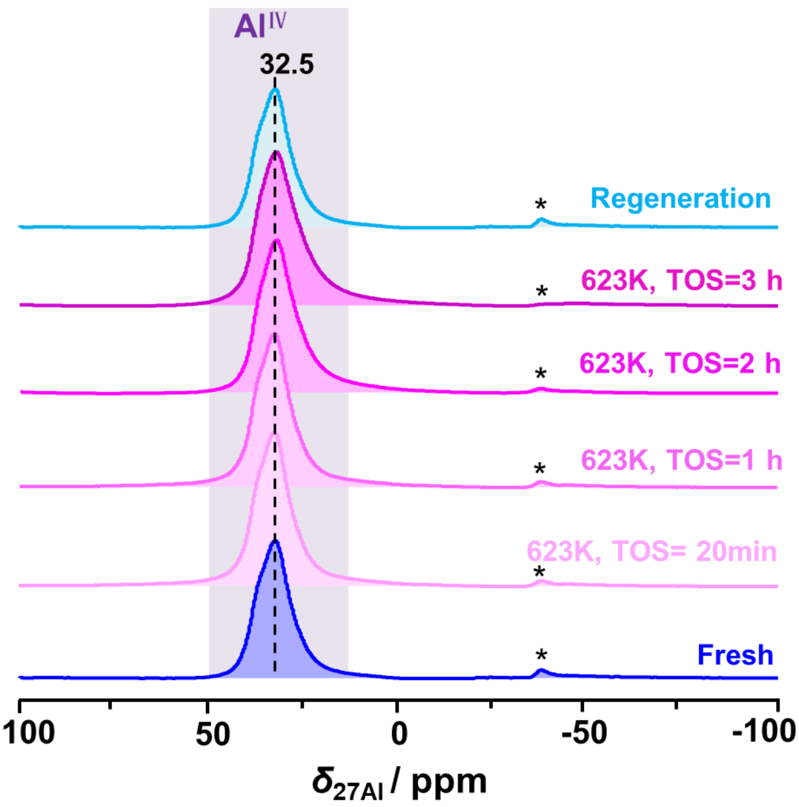

Supplementary Fig. 11 ^{27}Al MAS NMR spectra of the spent SAPO-34 catalysts obtained after MTO conversion at 623 K with different TOS.

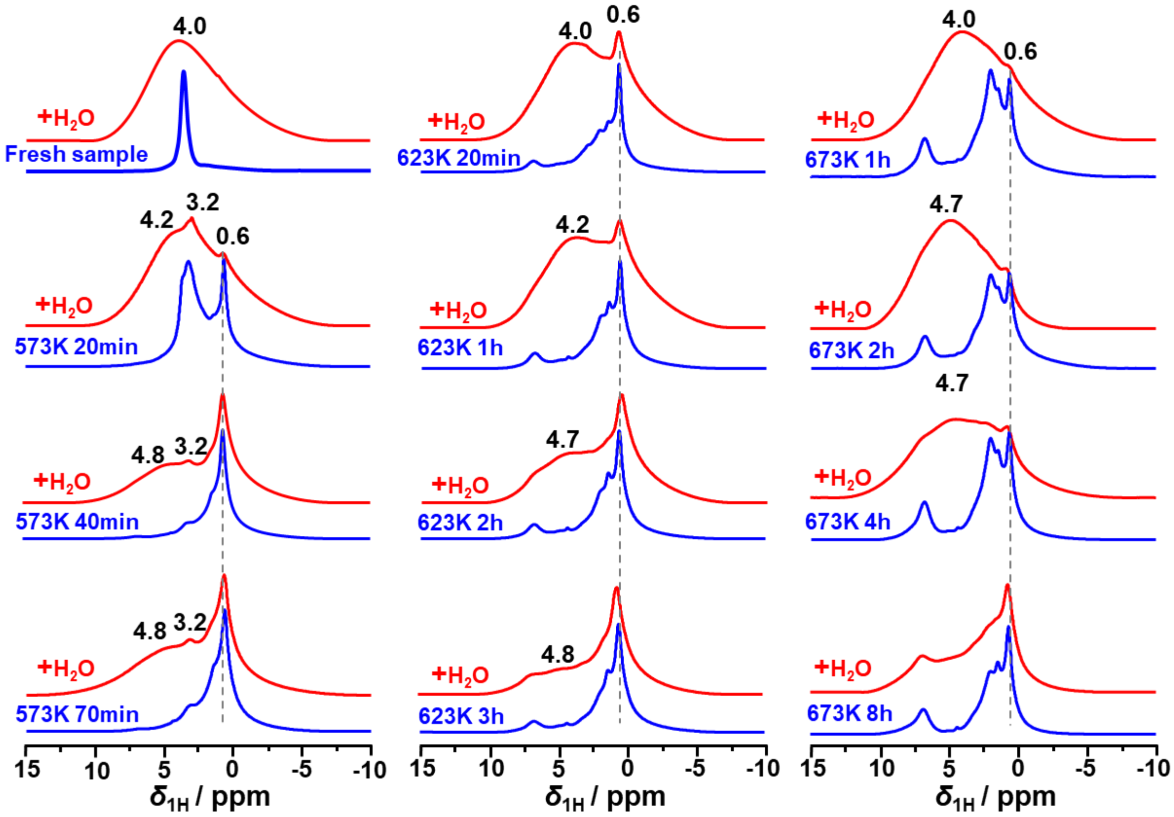

Supplementary Fig. 12 ^{1}H MAS NMR spectra of the fresh and spent SAPO-34 catalysts obtained after MTO conversion at different temperatures with different TOS, recorded before (blue) and after (red) water adsorption (with a hydration time of 24h).

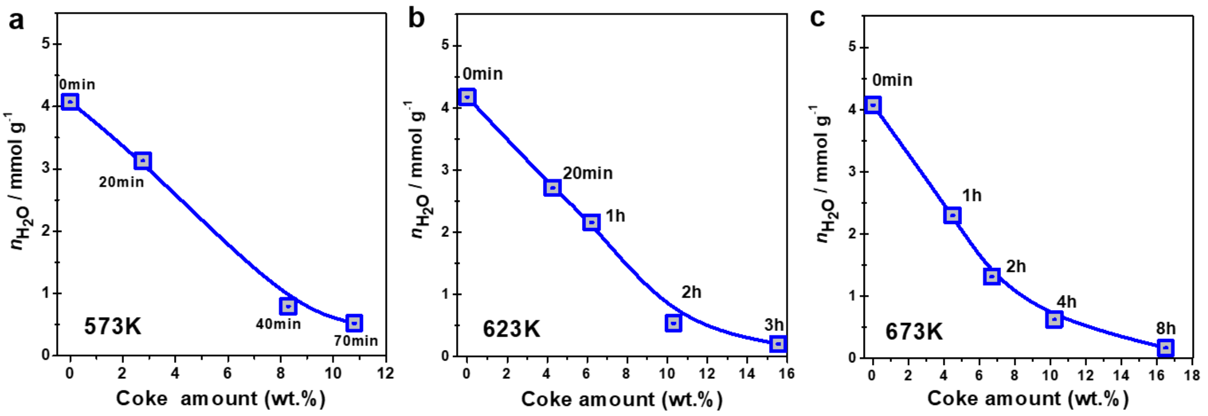

Supplementary Fig. 13 The number of adsorbed water molecules on fresh and spent SAPO-34 catalysts after MTO conversion at 573 (a), 623 (b) and 673K (c) with different TOS plotted as a function of the coke amounts.

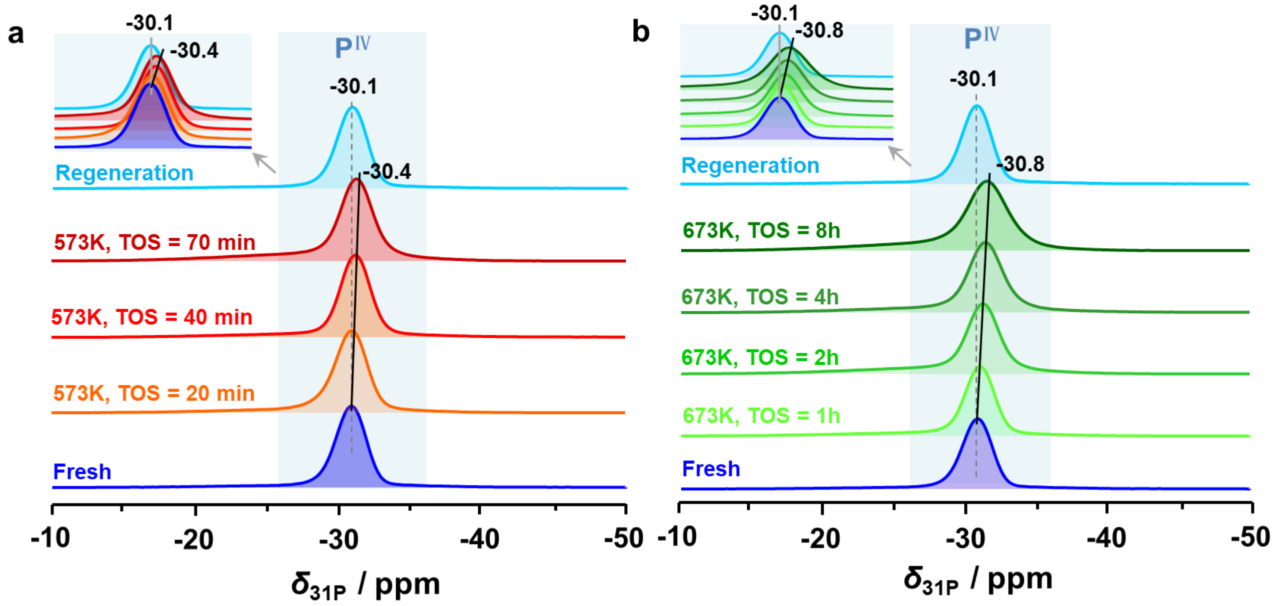

Supplementary Fig. 14 ^{31}P MAS NMR spectra of the fresh and spent SAPO-34 catalysts after the MTO conversion at 573 (a) and 673 K (b) for different TOS of 0-70 min and 0-8 h, respectively, under study.

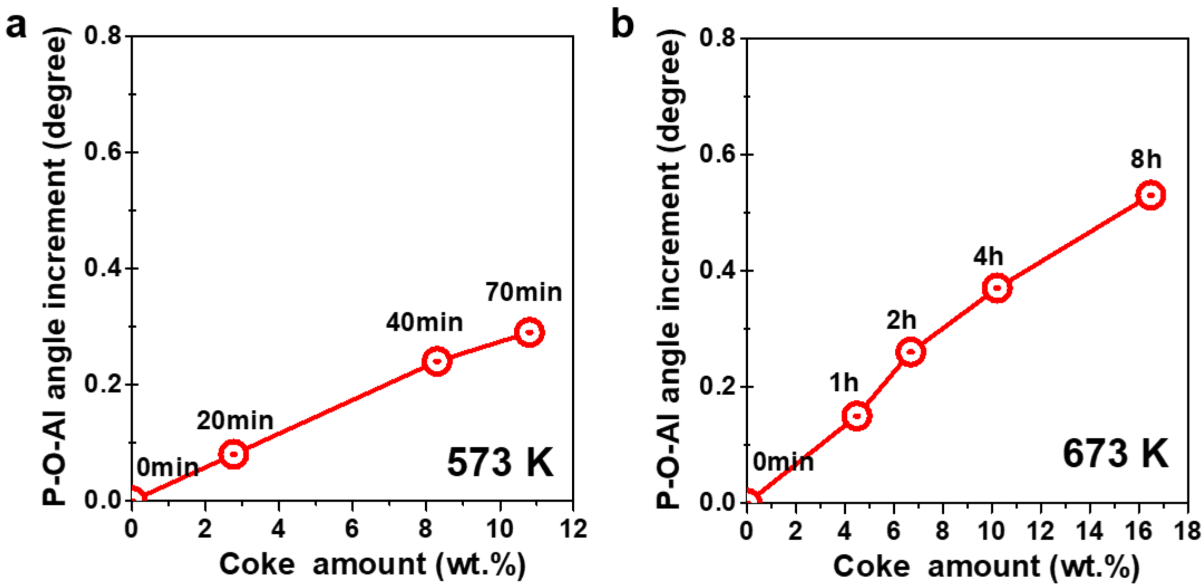

Supplementary Fig. 15 The increment of mean P-O-Al bond angles of spent SAPO-34 catalysts after MTO conversion at 623 (a) and 673 K (b) for different TOS.

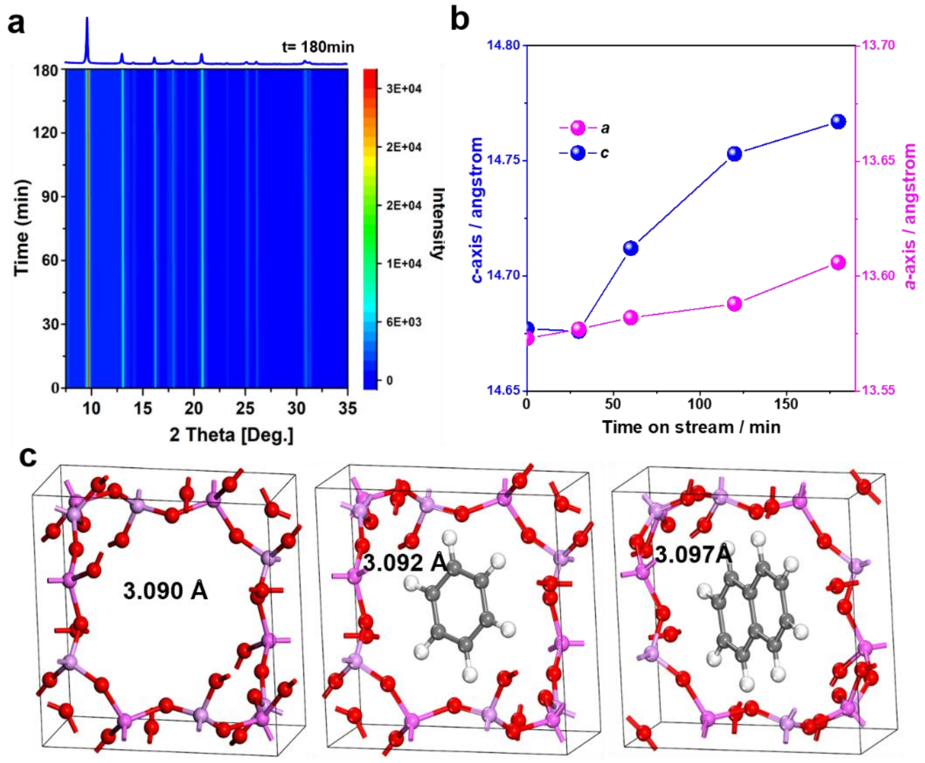

Supplementary Fig. 16 (a) In situ XRD patterns of SAPO-34 catalysts during the MTO conversion at 623 K with a TOS of 180 min. (b) Variation of the a- and c-axes of the selected SAPO-34 samples during the MTO conversion at 623 K with different TOS. (c) The mean theoretical distance of P-Al bond in the model SAPO-34 unit cell before and after the formation of different aromatics.

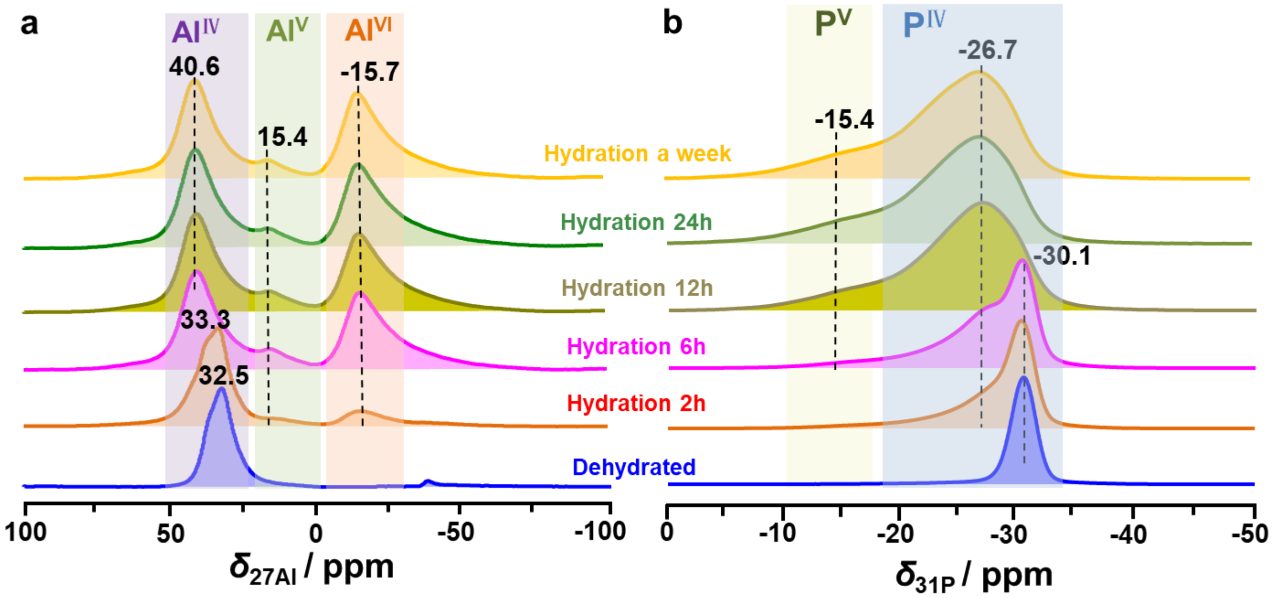

Supplementary Fig. 17 ^{27}Al (a) and ^{31}P (b) MAS NMR spectra of the fresh SAPO-34 catalyst after hydration at room temperature for different times.

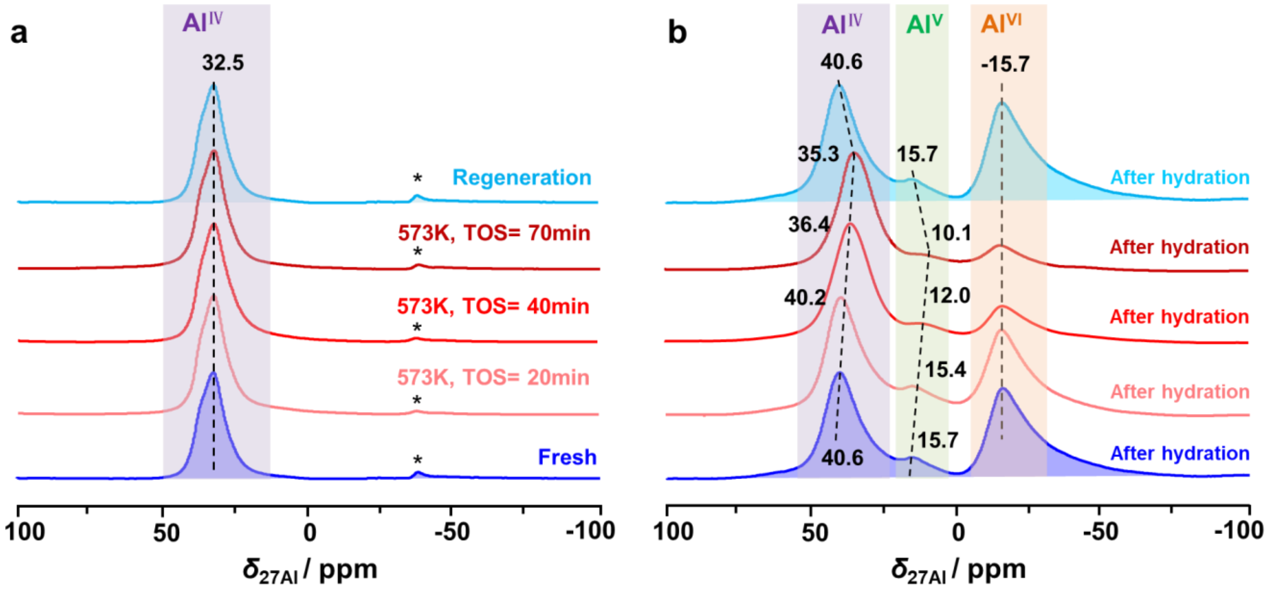

Supplementary Fig. 18 ^{27}Al MAS NMR spectra of the spent SAPO-34 catalysts obtained after MTO conversion at 573 K with different TOS of 0-70 min, recorded before (a) and after (b) hydration at room temperature for 24 h.

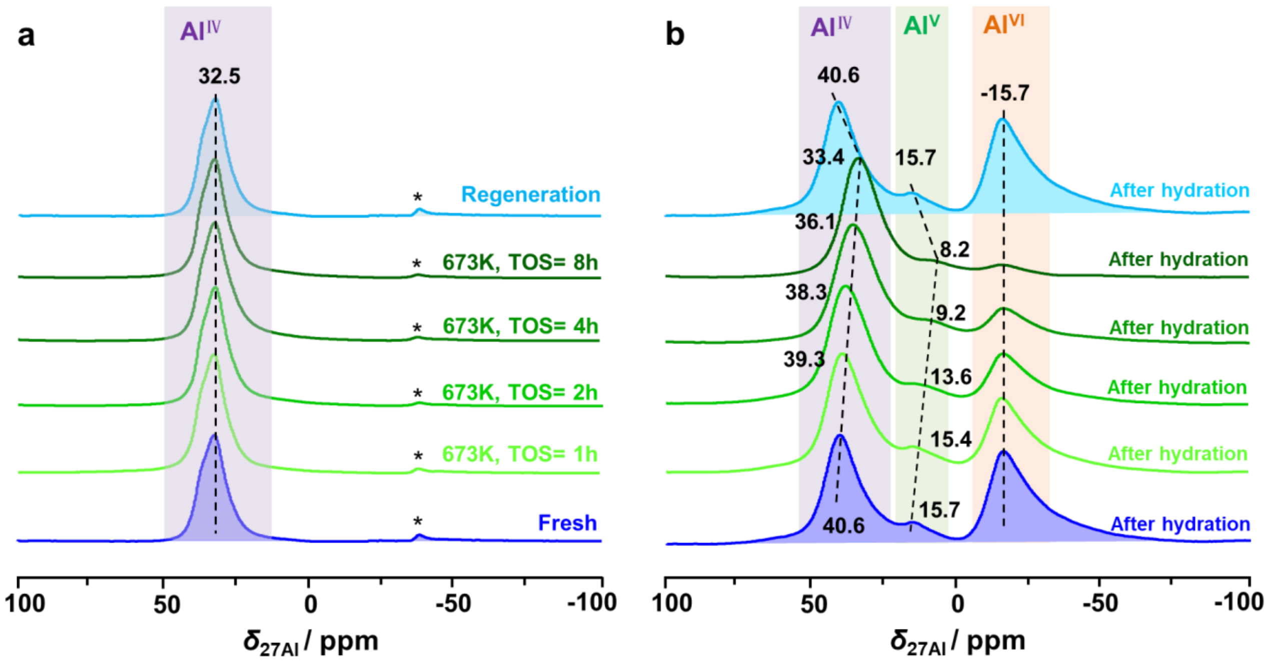

Supplementary Fig. 19 ^{27}Al MAS NMR spectra of the spent SAPO-34 catalysts obtained after MTO conversion at 673 K with different TOS of 0-8 h, recorded before (a) and after (b) hydration at room temperature for 24 h.

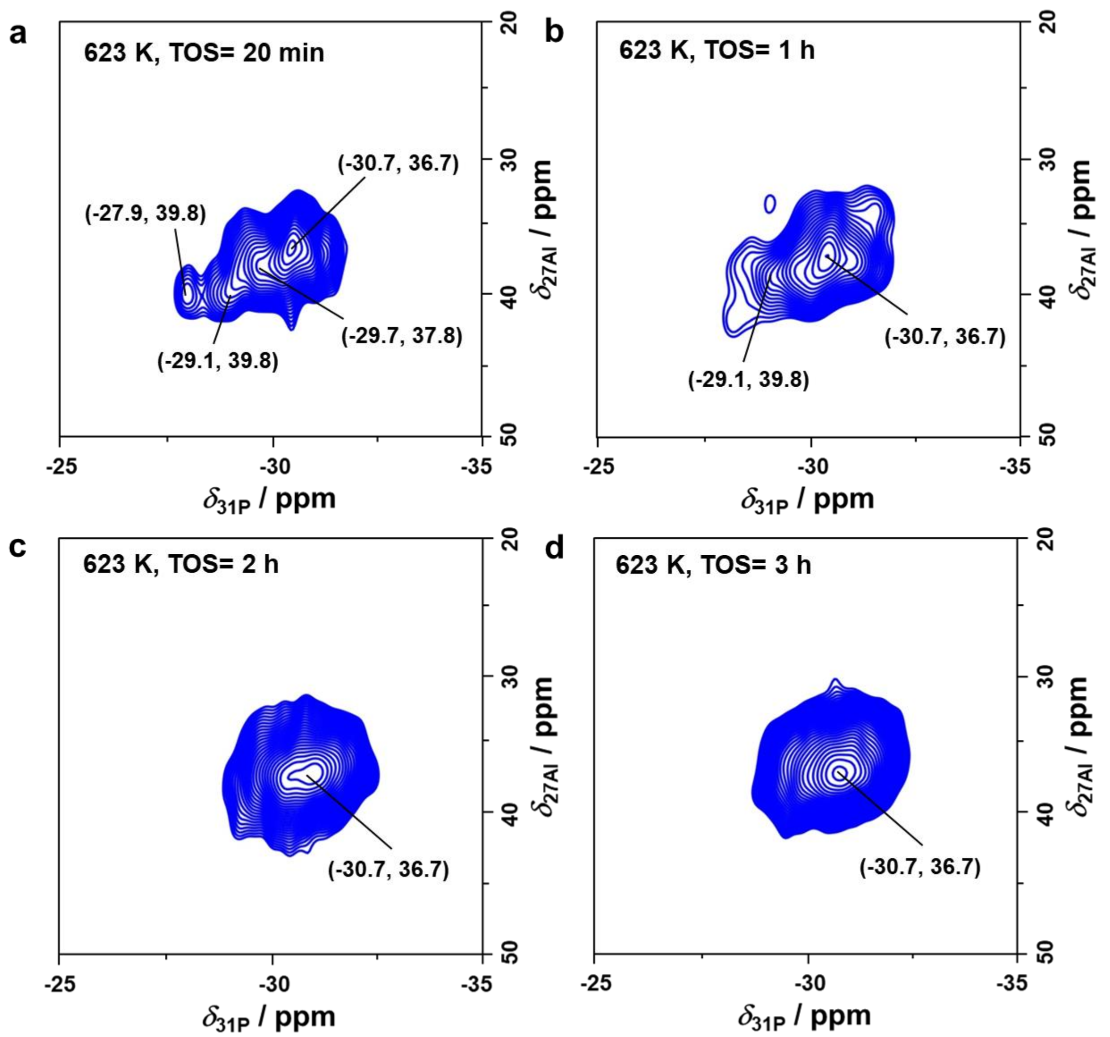

Supplementary Fig. 20 2D ^{31}P-^{27}Al HETCOR MAS NMR spectra of spent SAPO-34 zeolites after MTO conversion at 623 K for different TOS, recorded after hydration at room temperature for 24 h.

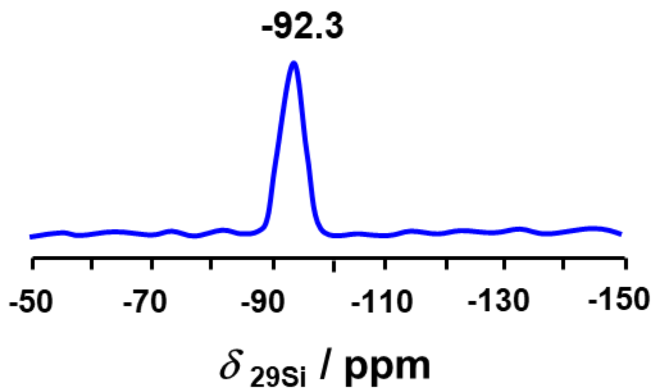

Supplementary Fig. 21 ^{29}Si MAS NMR spectra of the calcined SAPO-34 zeolite with the peak width of 8.6 ppm under study.

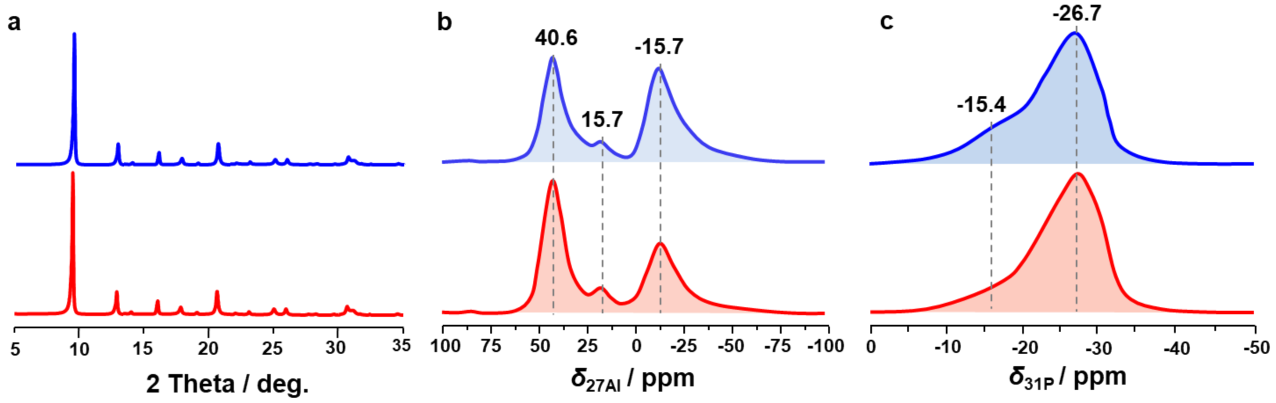

Supplementary Fig. 22 (a) XRD patterns, (b)^{27}Al and (c) ^{31}P MAS NMR spectra of the fresh (SA-34-F) and pre-coked SAPO-34 (SA-34-P) samples after steaming at 973 K for 30 h.

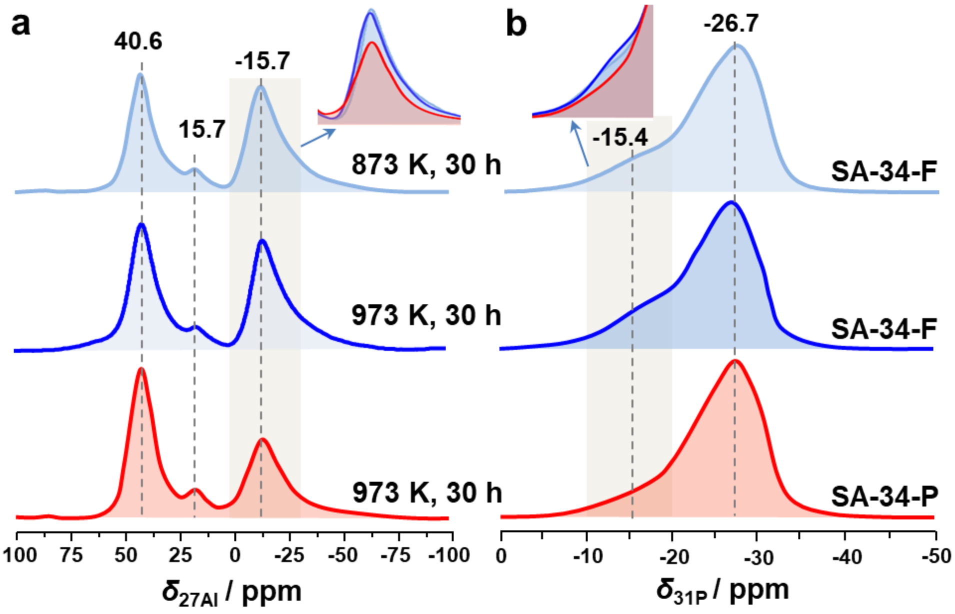

Supplementary Fig. 23 (a) ^{27}Al and (b) ^{31}P MAS NMR spectra of SA-34-F and SA-34-P samples after steaming at 873 and 973 K for 30 h.

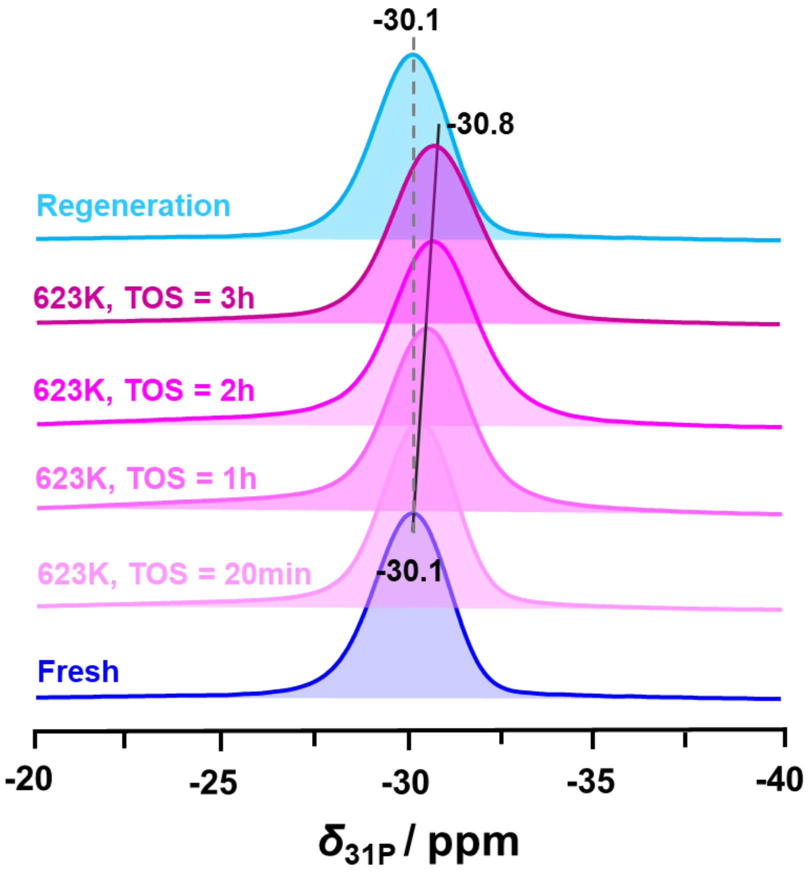

Supplementary Fig. 24 ^{31}P MAS NMR spectra of pre-streamed SAPO-34 catalysts after the MTO conversion at 623 K for different TOS of 0-3 h.

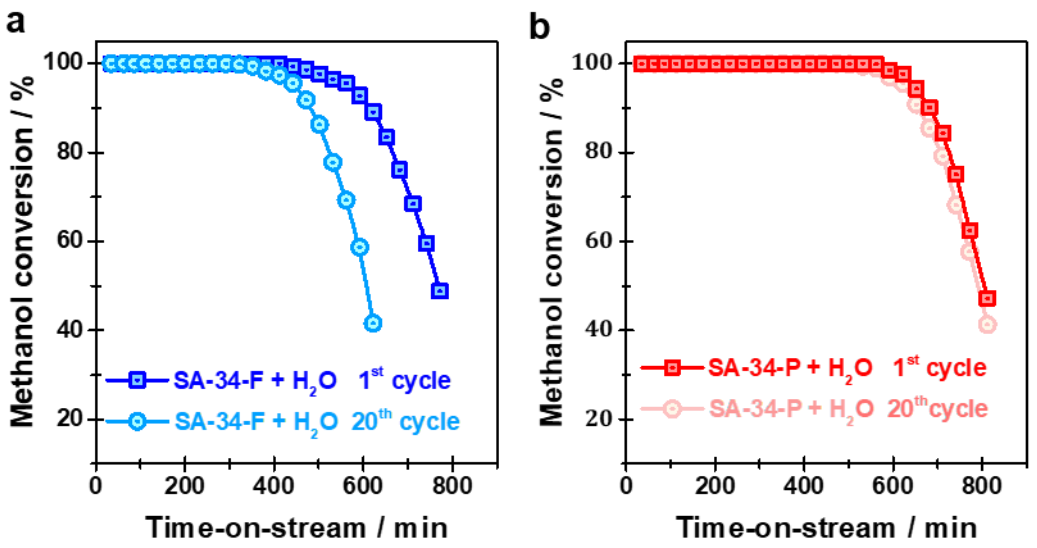

Supplementary Fig. 25 Methanol conversion over fresh (a) and pre-coked SAPO-34 (b) catalysts at 673 K with water co-feeding upon 20 cycles.

Supplementary Tables

Supplementary Table 1 Physicochemical properties of the calcined SAPO-34 sample under study.

Sample	Elemental analysis (mmol/g)[a]			Surface area[b] (m^2/g)	Micropore Volume[c] (m^3/g)	BAS[d] (mmol/g)
	n_{Al}	n_P	n_{Si}			
SAPO-34	6.94	6.46	0.72	553	0.28	0.51

[a] Determined by ICP. [b] Specific surface area obtained by the BET method. [c] Calculated from t plot.[d] Determined by ^1H MAS NMR spectroscopy and ammonia loading.

Supplementary Table 2 Assignments of the UV/Vis bands observed during the MTO conversion over SAPO-34 catalyst.

**Band at λ/nm**	**Assignments**	**References**
230-260	Dienes/unsaturated ketones	[1]
250-290	Neutral methylated benzenes	[1]
300-305	Monoenyl carbocations	[2,3]
335-340	Dienylic carbenium ions	[1,2]
370-390	Methylbenzenium ions	[4]
410-430	Methylnaphthalene/Trienylic/hi-ghly methylated arenium ions	[4,5-6]

Supplementary Notes

Supplementary Note 1

Effect of reaction temperature on MTO conversion and catalyst textural properties. For investigating the structure changes of this SAPO-34 catalyst during the MTO conversion, different reaction temperatures of 573-673 K were employed for comparison. Low MTO activity with an obvious induction period can be observed at 573 K (Supplementary Fig. 4). With increasing the reaction temperature to over 623 K, 100 % methanol conversion could be achieved. Simultaneously, large amounts of neutral polyalkylaromatics and the corresponding carbo-cations giving rise to the UV-vis bands at 260-300 and 370-390 nm, respectively, occurred. With further increasing the reaction temperature to 673 K, dienylic carbenium ions (335-340 nm), and neutral polyaromatics species and/or methylated naphthalene carbocations (425-430 nm) were formed as the dominant organic species. Simultaneously, an obvious increase of ethene selectivity could be observed, revealing that ethene is generated mainly from the aromatic-based cycle, in line with previous reports[7,8].

For catalyst deactivation at the low reaction temperature of 573 K, the catalyst deactivation should have another reason, as the BAS, the BET surface area and the micropore volume are preserved for the deactivated SAPO-34 catalyst (Supplementary Fig. 4c). According to previous studies[9,10], the catalyst deactivation at low reaction temperature could be due to the accumulation of saturated hydrocarbons, e.g. polymethyladamantane, as they could prevent the successive formation of active polyalkylaromatics. This is supported by the GC-MS studies, where lots of polymethyladamantanes were detected (Supplementary Fig. S8).

Supplementary References

1. Jiang, Y.; Huang, J.; Marthala, V.R. R.; Ooi, Y. S.; Weitkamp, J.; Hunger, M. *Microporous and Mesoporous Mater.* **105**, 132-139 (2007)
2. Kirisci,I.; Førster, H.; Tasi,G.; Nagy, J. B.; *Chem. Rev.* 99, 2085-2114 (1999)
3. M. J. Wulfers, F. C. Jentoft, *J. Catal.* **2013**, *307*, 204-213.
4. Dai,W.; Scheibe, M.; Guan,N.; Li,L.; Hunger, M. *ChemCatChem*, **3**, 1130-1133 (2011)
5. Mohan,J. Organic Spectroscopy Principles and Applications; Alpha Science International Ltd.: Harrow, 2002; pp 128, 137.
6. Park, J. W.; Lee, J. Y.; Kim, K. S.; Hong, S. B.; Seo, G. *Appl. Catal., A*, **339**, 36-44 (2008)
7. Svelle, S.; Joensen, F.; Nerlov, J.; Olsbye, U.; Lillerud, K. P.; Kolboe, S.; Bjørgen, M. J. Am. Chem. Soc. 128, 14770-14771 (2006)
8. Bjorgen, M.; Svelle, S.; Joensen, F.; Nerlov, J.; Kolboe, S.; Bonino, F.; Palumbo, L.; Bordiga, S.; Olsbye, U. J. Catal. 249, 195-207 (2007)
9. Wei, Y.; Li, J.; Yuan, C.; Xu, S.; Zhou, Y.; Chen, J.; Wang, Q.; Zhang, Q.; Liu, Z. *Chem. Commun.* **48**, 3082-3084 (2012)
10. Wei, Y.; Yuan, C.; Li, J.; Xu, S.; Zhou, Y.; Chen, J.; Wang, Q.; Xu, L.; Qi, Y.; Zhang, Q.; Liu, Z. *ChemSusChem*, **5**, 906-912 (2012)
